# Molecular pedigree reconstruction and estimation of evolutionary parameters in a wild Atlantic salmon river system with incomplete sampling: a power analysis

**DOI:** 10.1186/1471-2148-14-68

**Published:** 2014-03-31

**Authors:** Tutku Aykanat, Susan E Johnston, Deirdre Cotter, Thomas F Cross, Russell Poole, Paulo A Prodőhl, Thomas Reed, Ger Rogan, Philip McGinnity, Craig R Primmer

**Affiliations:** 1 Division of Genetics and Physiology, Department of Biology, University of Turku, Turku, Finland; 2 Marine Institute, Furnace, Newport, Co., Mayo, Ireland; 3 Aquaculture & Fisheries Development Centre, School of Biological, Earth & Environmental Sciences, University College Cork, Cork, Ireland; 4 Institute for Global Food Security, School of Biological Science, Medical Biology Centre, Queen’s University, Belfast, Northern Ireland; 5 Present address: Institute of Evolutionary Biology, University of Edinburgh, West Mains Road, Edinburgh, UK

**Keywords:** *Atlantic salmon*, Heritability, Incomplete sampling, MasterBayes, Parentage assignment, Power analysis, Reproductive success

## Abstract

**Background:**

Pedigree reconstruction using genetic analysis provides a useful means to estimate fundamental population biology parameters relating to population demography, trait heritability and individual fitness when combined with other sources of data. However, there remain limitations to pedigree reconstruction in wild populations, particularly in systems where parent-offspring relationships cannot be directly observed, there is incomplete sampling of individuals, or molecular parentage inference relies on low quality DNA from archived material. While much can still be inferred from incomplete or sparse pedigrees, it is crucial to evaluate the quality and power of available genetic information *a priori* to testing specific biological hypotheses. Here, we used microsatellite markers to reconstruct a multi-generation pedigree of wild Atlantic salmon (*Salmo salar* L.) using archived scale samples collected with a total trapping system within a river over a 10 year period. Using a simulation-based approach, we determined the optimal microsatellite marker number for accurate parentage assignment, and evaluated the power of the resulting partial pedigree to investigate important evolutionary and quantitative genetic characteristics of salmon in the system.

**Results:**

We show that at least 20 microsatellites (ave. 12 alleles/locus) are required to maximise parentage assignment and to improve the power to estimate reproductive success and heritability in this study system. We also show that 1.5 fold differences can be detected between groups simulated to have differing reproductive success, and that it is possible to detect moderate heritability values for continuous traits (h^2^ ~ 0.40) with more than 80% power when using 28 moderately to highly polymorphic markers.

**Conclusion:**

The methodologies and work flow described provide a robust approach for evaluating archived samples for pedigree-based research, even where only a proportion of the total population is sampled. The results demonstrate the feasibility of pedigree-based studies to address challenging ecological and evolutionary questions in free-living populations, where genealogies can be traced only using molecular tools, and that significant increases in pedigree assignment power can be achieved by using higher numbers of markers.

## Background

Pedigree reconstruction provides a robust framework for the study of population evolutionary dynamics in the wild [1,2]. By inferring the degree of relatedness among individuals within and between generations, it is possible to infer the survival and reproductive success of individuals, which, in turn, allows for the testing of a wide range of hypotheses relating to the demographic and evolutionary trajectories of populations and species over several generations. Furthermore, when used in combination with specific mixed-effects model statistical approaches [3-5], pedigree information allows for the estimation of relevant quantitative genetic parameters, including additive genetic and environmental variances and covariances for traits, as well as their underlying and associated quantitative trait loci (QTL). Thus, many empirical studies have successfully utilised pedigree information derived from physical tagging, genetic data or a combination of both approaches, to test a wide range of evolutionary and ecological hypotheses in the wild for various taxa [2,6]. Such studies have provided insights on the long term response of populations to environmental change [7], the effect of inbreeding depression in small or introduced populations [8,9], the elucidation of mating systems [10,11], the estimation of the variance components of life history traits [12,13], and the detection of QTLs [14,15]. However, in species with external fertilisation and limited post-hatching parental care, the construction of pedigrees through observational means is limited by the inability to physically mark individuals and/or monitor parent-offspring associations. In addition, in cases where direct observations are possible, they may be subject to additional error such as those resulting from extra pair paternity as commonly shown in birds (e.g. see [16]). Therefore, the use of genetic information to reconstruct pedigrees emerges as a viable alternative to extract genealogical information within wild populations.

Atlantic salmon (*Salmo salar*) is an economically important species, with many wild populations subject to long-term population monitoring and biological sample collection spanning many decades. In principle, given the high level of homing to their natal rivers prior to reproduction, retrieving pedigree information in salmon is more achievable compared to many other fish species. Indeed, pedigree reconstruction in salmon and other salmonids based on DNA genotyping has been shown to be both feasible and valuable for conservation biology and evolutionary research [17-23]. Thus, pedigree-based approaches have been successfully used to address important questions in conservation biology, particularly in determining the efficacy of supplementing wild populations with captive bred individuals [24]; a number of these studies have demonstrated that captive bred salmon may have reduced reproductive success in the wild e.g. [19,21-23,25,26], but see [27]. Pedigree reconstruction in salmon is also important for estimating quantitative genetic parameters in wild populations. However, to date, the majority of quantitative genetic data in salmonids have been derived from common garden experiments in artificial environmental settings (for a review, see [28]). Whilst clearly providing valuable information of relevance to salmonid biology, the insights into population evolutionary dynamic processes provided by these data may be only of limited value when extrapolating to wild populations, due to the effect of variable environments on both additive genetic and environmental components of trait variance [6,28-30]. To date, only a handful of studies have estimated quantitative genetic parameters of fitness-related traits in the wild e.g. [17,31-33]. This indicates that additional efforts to reconstruct pedigrees in wild salmonid populations are required to fill this critical knowledge gap.

Despite the great potential for pedigree-based studies in wild salmonid populations, there are a number of possible problems that need to be considered. First, the sampling of potential parents in any given year is often incomplete. Partial sampling regimes may be due to several factors, such as logistical constraints (e.g. very large and/or complex aggregations of fish) or issues associated with the handling of wild fish, where possible impacts on behaviour or subsequent survival must be considered. Second, precocious male parr (i.e. males that mature in fresh water before going to sea and potentially, if they survive, returning to spawn second time) can be important components of breeding populations [34,35] which may remain unsampled if only sea migrating fish that have not matured in freshwater previously are sampled. Finally, long-term datasets that may allow multi-generation pedigree reconstruction are often based on archived material. Depending on the storage conditions, DNA extracted from archived material is likely to be of varying quality, with older samples often more problematic as DNA quality tends to deteriorate with sample age e.g. [36]. Given these many variables, it is imperative that power analysis type methods e.g. [13] are conducted in the early stages of project development. In the context of pedigree reconstruction, power analysis allows for the exploration of the best approaches to obtain reasonably accurate, unbiased parentage assignments and parameter estimates within the given logistical and budget constraints of a particular study.

**Figure 1:**
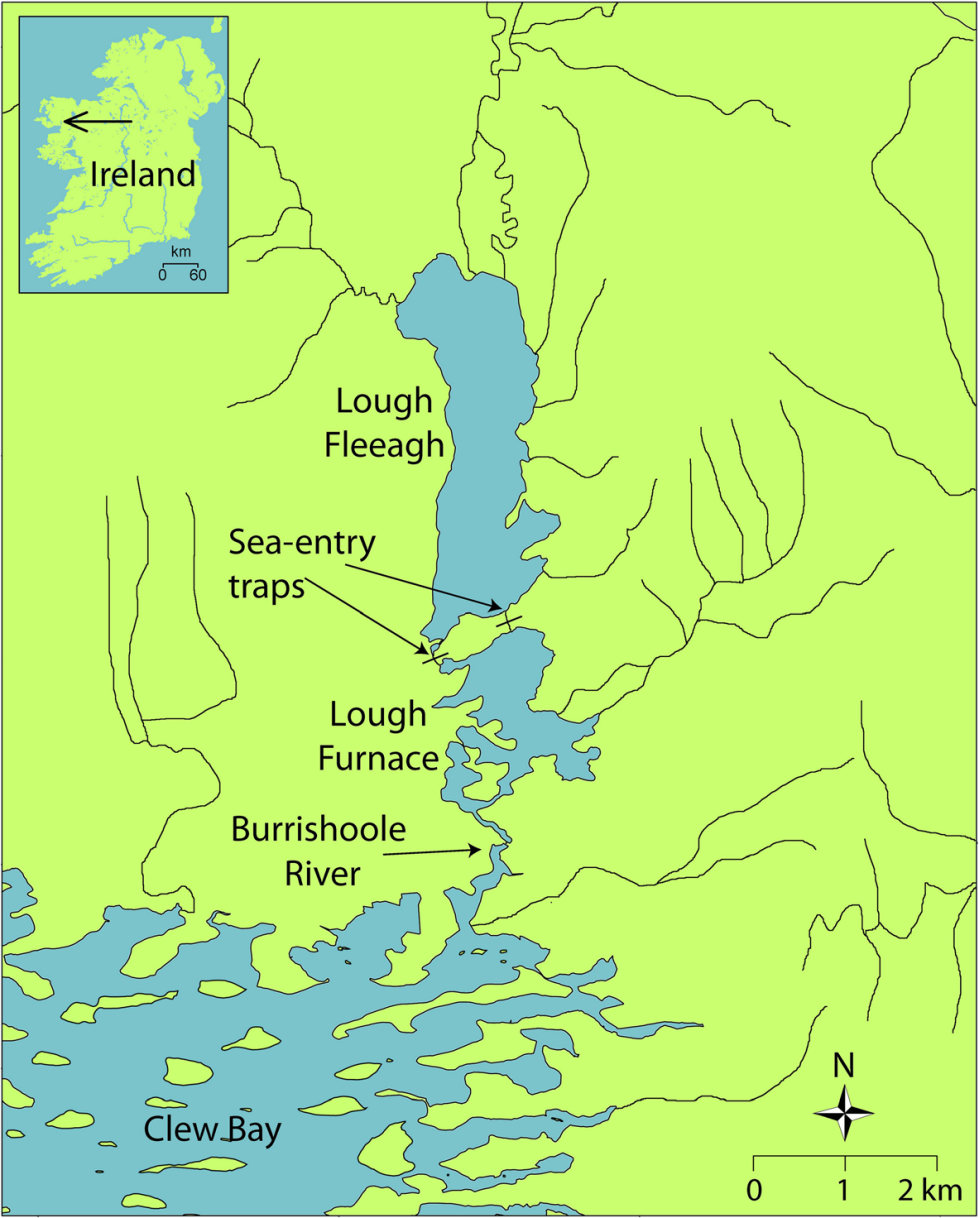
**Map of the Burrishoole River system in Ireland.** Two traps in which fish going upstream are monitored, and fish going downstream are sampled, are indicated with arrows.

**Figure 2:**
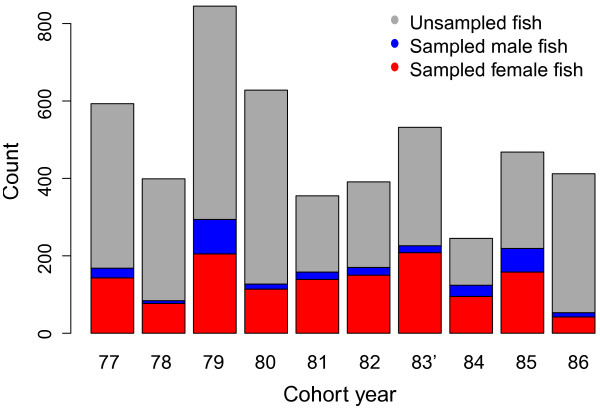
**Census and sampling numbers among cohorts.** Bar plot showing total number of wild returning fish and sex-categorised sampled portions in 10 consecutive cohorts of the Burrishoole Atlantic salmon analysed in this study.

Atlantic salmon of the Burrishoole River system on the west coast of Ireland have been consistently monitored since 1956, with complete annual censuses conducted since 1969 [25,37-39] (Figure 1). A captive breeding programme was established near the mouth of the river system in the early 1960s, using fish collected from the same system [25]. Since then, variable proportions of hatchery-origin adult fish have migrated into the system (either intentionally or unintentionally) and supplemented the wild spawning population. The entrance to the system is spanned by two fish traps through which all sea-bound or sea-returning fish must pass; tissue samples (scales) and length measurements are collected from all hatchery-reared adult fish on their upstream migration, and from wild fish on their downstream migration some 3–4 months after reproduction. Because of this consistent long-term monitoring and well-recorded management history (Figure 2, Table 1), the Burrishoole system provides an ideal benchmark for investigating the effects of hatchery supplementation on the biology, ecology and genetics of wild *S. salar*. Indeed, this useful information has already been exploited in a number of previous studies examining, for instance, the fresh water and marine age composition in wild and hatchery reared groups [38], physiology [40,41], the fresh water performances of wild and hatchery fish e.g. [25,42], and migration behaviour [43]. Despite these previous studies, there remains a limited understanding of some key biological parameters including the mating system, lifetime reproductive success and survival dynamics over generational periods. Thus, the pedigree reconstruction of the Burrishoole *S. salar* should allow a better understanding of such parameters.

**Table 1:** **Sampling statistics of the *S. salar *** **cohorts included in the study**

**Cohort year**	**Number of wild returning fish**^**1**^	**Number of sampled fish**^**2**^	**Total number of analysed fish**^**3**^	**% of sampled fish analysed**	**Overall % of samples analysed**
1977	594	169	143	84.6	24.1
1978	400	85	68	80.0	17.0
1979	854	303	273	90.1	32.0
1980	628	127	124	97.6	19.8
1981	355	158	152	96.2	42.8
1982	392	171	150	87.7	38.3
1983	533	227	212	93.4	39.8
1984	245	124	124	100.0	50.6
1985	472	223	217	97.3	46.0
1986	412	53	52	98.1	12.6
Total	4885	1640	1515		
Mean	488.5	164	151.5	92.5	32.3

^1^Number returning fish includes all returning wild individuals from the sea, but excluding precocious male parr.

^2^Number of sampled fish includes all post-spawned wild adults returning to sea (kelts) after breeding season.

^3^Total number of analysed fish includes all sampled fish that were successfully genotyped with ≥ 7 microsatellite loci.

In this study, we have evaluated the feasibility of a pedigree-based approach for exploring population biology and stock dynamics in the Burrishoole River Atlantic salmon. First, we performed a simulation and sensitivity analysis to detect the number of microsatellites required for maximising successful parentage assignment, given the available molecular markers, incomplete sampling regime and the variable quality of DNA in the samples available. Second, we estimated the power to detect variation in reproductive success among different groups of breeders. Finally, we estimated the detection power and confidence intervals of heritability estimates using simulations based on the empirical sampling and pedigree structure, and estimated the power gain in heritability and reproductive success detection when using higher number of markers.

## Methods

### Study system and sampling

**Figure 3:**
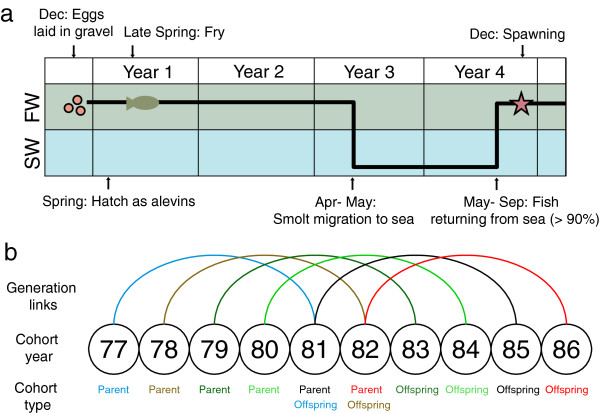
**Burrishoole Atlantic salmon life cycle. (a)** The complete life cycle indicating all possible generational links. **(b)** Presumed generational links within the sampling period.

The Burrishoole River system is situated in NW Ireland (53°59^′^ N 09°37^′^ W; Figure 1); the largest lake in the catchment (Lough Feeagh) has a surface area of 3.9 km^2^. *S. salar* in the Burrishoole River system are anadromous, typically returning to fresh water between June and September to spawn in December. The majority (i.e. 90%) of juveniles spend two and a half years in fresh water followed by one year at sea (1SW), after which they return to fresh water to spawn [44] (Figure 3a). The remaining 10% of adults spend one additional year at sea before returning to spawn in fresh water as two sea winter fish (2SW; Figure 3a). In addition, precocious male parr may account for up to 30% of the breeding males [45]. A total trapping system for the counting of returning adults and migrating smolts has operated at Burrishoole since 1969 at two locations above the tide (Figure 1), allowing the quantification of all fish entering and leaving the system. All individuals entering and leaving Lough Feeagh must enter one of two traps where they can be counted and sampled before entering or leaving the system (Figure 1). The number of wild salmon spawning in the system during the ten years period considered in the present study ranged from 245 in 1984 to 854 in 1979, with a mean number of 489 individuals per cohort year (i.e. run year; Table 1).

Post-spawn wild individuals (i.e. kelts) were sampled on their downstream migration (March – May). Sampled individuals were sexed and measured for length (± 0.25 cm) and three to five scales were removed and archived in individual vanilla paper scale envelopes. Sex was determined on the basis of secondary sexual characteristics (principally the distinct elongation of the lower jaw in male fish known as a kype), which are readily discerned in post-spawned individuals. The current study utilised archived scales from ten consecutive cohorts collected between 1977 and 1986 (Table 1). This encompassed six discrete sets of parent-offspring cohort pairs, enabling family relationships and associated life history patterns to be established between linked cohorts (1977–1981, 1978–1982, 1979–1983, 1980–1984, 1981–1985 and 1982–1986), including two three-generational inter-related pedigrees (1977-1981-1985 and 1978-1982-1986; Figure 3b).

A total of 1640 fish were sampled (Figure 2), of which 1515 (92.5%) were successfully genotyped for at least seven microsatellite loci (Table 1, Figure 2), with the proportion of analysed individuals in each cohort ranging from 12.6% to 50.6% (Table 1, Figure 2). Assuming a four-year generation time between cohorts, the average reproductive success per cohort, calculated as the ratio of progeny to parental census size, also varied across years (Table 1). The sampling was highly skewed towards females as a result of higher male mortality associated with spawning, with female and male individuals comprising 81.2% and 17.8% of sampled proportion per cohort, respectively (Figure 2). The Burrishoole river is the national (Government of Ireland) index river for research into Atlantic salmon and operates under licence (Fisheries Acts 1959–2003) from the Department of Agriculture, Food and Marine and by permission of the Minister of Agriculture, Food and Marine. All sampling was carried out under authorisation (Sec. 4) of the Fisheries Acts, 1959 to 2003.

### DNA genotyping and genetic diversity estimation

DNA was extracted from scale samples (two scales per fish) using a QIAamp mini kit (Qiagen Inc. Valencia, CA, USA) and 5 μl of proteinase K (0.5 mg/ml final concentration). The extraction was performed following the manufacturer’s instructions for DNA extraction from tissues, except that the elution volume was reduced to 100 μl instead of the suggested 200 μl in order to increase the DNA concentration. Each fish was genotyped at 14 microsatellite markers (ave. 12 alleles per locus, ave. Ho = 0.70) known to amplify reliably in archived scale samples [46]. Further, they can be electrophoresed in a single semi-automated sequencer column (denoted as Panel 1 markers; Additional file 1) and amplified in two multiplexed PCR reactions (MP1 and MP2; Additional file 1). Four cohorts (1977, 1979, 1981 and, 1983) were further amplified at an additional 15 markers (Panel 2; Additional file 1), to be used in the heritability simulation and to further assess potential improvement in power through the use of higher marker number (see below for details). The sets of loci used for PCR multiplexing have earlier been optimised for use with scale-extracted DNA in a highly efficient manner, which yields high genotyping success with low error rates [46] and therefore provide an empirical basis for assessing optimal microsatellite number. The details of all primers, PCR reactions and genotyping information are given in Additional file 1. For fragment analysis, 10 μl of HiDi and 0.1 μl of GSL600 LIZ size standard (Life Technologies. Norwalk, CT, USA) were mixed with 2 μl of the pooled PCR multiplex dilution (see details in Additional file 1), and microsatellite fragments were separated using an ABI 3130xl Prism Genetic Analyzer. Genotyping was carried out with Genemarker 2.2 (Softgenetics) software using custom-made allele bins, and the allele scorings were visually inspected and edited.

Unless otherwise stated, all statistical analyses were performed using R software version 2.15.2 [47]. Genetic diversity indices, including allelic richness, number of alleles, observed and expected heterozygosities, and the polymorphism information content (PIC) within cohorts, were calculated using the PopGenKit package 1.0 [48]. Within-sample deviations from Hardy-Weinberg equilibrium (HWE) were estimated using the *HWE.test.genind* function (with 100000 permutations) of the adEgenet package 1.3-6 [49]. Null allele frequencies were estimated using cervus 3.0 [[50]]. Allele frequencies were estimated for every combined parent-offspring cohort pair, both in parentage inference as well as when simulating genotypes, whereby no differentiation was assumed between parental and offspring cohorts. Therefore, we assessed temporal stability of the Burrishoole Atlantic salmon using genetic differentiation statistics *θ*_*ST*_[51] and Jost’s D [52] with the diveRsity package 1.5.0 [53] and by bootstrapping loci 10000 times. For Jost’s D, we measured differentiation on a per locus basis (i.e. single locus estimate) as suggested in [54], since it is sensitive to mutation rate differences across loci [55].

The exclusion probability of a marker, which describes the proportion of unrelated parental candidates expected to be excluded by a marker, was calculated using the formula:

$$\begin{array}{c} \mathrm{exclusion}\;\mathrm{probablit}y_{j}={\left[\Sigma_{i-1}^{N}1-\left(2p_{\mathrm{ji}}-p_{\mathrm{ji}}^{2}\right)\right]}^{2}\\ \;+\Sigma_{i-1}^{N}\left[1-{\left(2p_{\mathrm{ji}}-p_{\mathrm{ji}}^{2}\right)}^{2}\right]. \end{array}$$

where *p*_ji_ is the allele frequency of the *i*^th^ allele of locus *j*. Although exclusion probability is not fully informative in models with genotyping error rate, it still provides a rough estimate of parentage assignment power on a per-locus basis and across all loci.

### Parentage analysis and evaluation of marker number by simulations

We used a Bayesian approach in constructing the pedigrees as implemented in the MASTERBAYES package 2.50 [56]. MASTERBAYES incorporates the estimation of genotyping error rate and the number of unsampled individuals when inferring pedigree structure, which provides better assignment rates especially if unsampled individuals represent a considerable proportion of the potential parent set [56,57]. In the pedigree constructions, we assumed a four year interval between parent and offspring cohorts, such that an individual within a cohort was defined as a potential parent for any individual sampled four years later (Figure 3b).

Individuals successfully genotyped at fewer than seven of the 14 Panel 1 markers (7.6% of all individuals) were excluded from the parentage analyses (Table 2, Additional file 2). The allelic dropout (E1) and stochastic genotyping error rates (E2), based on Wang’s error rate model [58], were estimated within the MASTERBAYES framework based on the repeat genotyping of a proportion of the individuals in the dataset [56]. Using the identity analysis in cervus, we detected a total of 105 near identical individuals, which included 99 pairs and 6 triplicates that had been genotyped more than once. Nineteen of these were technical replicates (i.e. the same individual genotyped more than once using the same DNA isolate) and 86 were biological replicates, in which DNA was isolated from different scales that had been sampled at different times (i.e. individuals that were captured more than once in the same year, or in the subsequent year (5% of the total number of individuals sampled. See: Table 1)). The error rates were estimated on a per-locus basis and across a temporal gradient, since both locus and time were important determinants of the error rate (Additional file 3). In subsequent analyses, error rate probabilities were parameterized individually based on cohort membership.

**Table 2:** Summary of descriptive statistics of the microsatellite marker panels used in the study

	**Panel 1 markers (*N*** **= 14)**	**Panel 2 markers (*N*** **= 15)**
Total number of individuals	1640	832
Total number of genotyped individuals	1436	726
% Mean genotyping success	88.9	87.2
Mean allele number	12	11.9
Mean allelic richness	10.2	11.4
Mean *H*_*O*_	0.697	0.661
Mean *H*_*E*_	0.725	0.684
Null allele frequency	0.021	0.018
Mean PIC	0.7	0.65
Combined exclusion probability	0.999942	0.999688

Ho = observed heterozygosity, Ho = expected heterozygosity, PIC: polymorphism information content.

MASTERBAYES was also used to estimate the number of unsampled parents within a Bayesian framework [56,57,59]. We first estimated the priors for the number of unsampled parents based on census size information (Table 1) and the sex ratio. As females generally make up 55 to 70% of the salmon in the system, the prior used for unsampled females was 55 to 70% of the census size sampled from a uniform distribution. Likewise, the standard deviation for the number of unsampled females was estimated from the above distribution, but with a value three times larger to cover a broader parameter space in the random walk. Within males, precocious male parr may account for up to 30% of breeding males [45], which we accounted for in the unsampled male prior. We specified a mean of 17% mature male parr within all breeding males in the prior estimate with a high sampling variance of distribution. In the simulations, the priors were selected for a gamma distribution with shape and scale parameters of 20 and 0.8, respectively. (Scale parameter 20 is equivalent to 17% contribution of precocious males to the male breeding pool, while the shape parameter 0.8 makes the distribution slightly skewed to the left.) The standard deviation for the unsampled male number was estimated based on the above distributions, and a standard deviation ten times higher was used in the analysis to cover a much broader parameter space in the random walk and account for the higher uncertainty associated with the precocious male parr contribution.

### Genotype simulation

Genotypes were simulated to generate hypothetical, but realistic, pedigrees in order to assess optimal marker number as well as for use in heritability power analyses. We simulated genotypes based on empirical data from the Burrishoole system using the *simgenotype* function in the MASTERBAYES package, based on the empirical allele frequency distributions of Panel 1 markers, and by defining the relationships among individuals based on the empirical parentage assignment using 14 markers (see below) with a low probability threshold (p = 0.50) to define a higher number of parental links. The genotyping error rate and missing genotype frequency were also parameterised based on the empirical results estimated above; for every simulated genotype, error rates were sampled from the posterior probability distribution. Six different marker numbers (5, 10, 14, 20, 28 and 50 loci per individual) were generated, and each pedigree associated with each of the six separate parent-offspring cohort pairs was simulated 100 times. The markers were generated randomly (with replacement) based on the empirical allele frequency distributions of Panel 1 markers, assuming independent assortment.

### Parentage analysis of simulated genotypes

The parentage assignment in the simulated parent-offspring cohort pairs was conducted in MASTERBAYES with 13,000 iterations, 3,000 burn-ins and a thinning interval of 10. The mismatch tolerance for the initial data filtering was adjusted to two loci for all simulations except with 50 loci, where the mismatch tolerance was adjusted to three. Other parameters (e.g. priors for the unsampled male and female numbers, error rates) were the same as above. Metropolis Hasting acceptance rates were in the range of 25% - 35%, after tuning scaling constants to 0.5 and 0.1 for unsampled dam and unsampled sire, respectively, using the *tunePed* function in MASTERBAYES. Likewise, autocorrelation of Markov Chain was monitored in all analyses and was reasonable (r^2^ < 0.06) in all runs at respective thinning intervals. Next, most potential parents were accepted using one of five Bayesian posterior probability thresholds (p = 0.2, 0.4, 0.6, 0.8, and 0.95), and pedigrees were constricted based on the inferred parent-offspring relations [56]. For every pedigree generated, we compared it to the simulated (true) pedigree and calculated the number of true and false assignments. Further, we explored the effect of parameterising missing genotypic information and genotyping error rates in the simulation on the accuracy of parentage assignment efficiency. For that purpose, we sequentially excluded these parameters in simulating genotypes, such that no missing genotypes were assumed or both error rates (ER1 and ER2) were assumed to be zero during the simulations (MASTERBAYES, *simgenotype* function), and the parentage analysis was otherwise performed as above. Finally, we attempted to use colony2 software for parentage and halfsib inference [60], but the initial results suggested prohibitively long run times would be required to resolve parentage. Likewise, accurate half-sib inference appeared to be unlikely, probably due to run time limitations or as the result of smaller half sib groupings.

### Empirical pedigree reconstruction

We assigned parentage in six parent-offspring cohort pairs (with 14 Panel 1 markers) using the MASTERBAYES package (130,000 iterations, 30,000 burn-ins and a thinning interval of 50, mismatch tolerance = 2) and by using highly stringent (0.95) and less stringent (0.8) Bayesian posterior probability thresholds. The higher threshold is a commonly used threshold in the literature for the MASTERBAYES package e.g. [56,57], while the lower threshold was selected arbitrarily.

Additionally, we used both Panel 1 and Panel 2 (i.e. 29 loci in total) in assigning parentage in a subset of parent-offspring cohort pairs (1977–1981 and 1979–1983). This further analysis was used to determine the detection power of heritability estimations (see below) and to compare empirical and simulated parentage efficiency.

### Estimating the power to detect differences in relative reproductive success (RSS) among groups of individuals

Within a cohort, we simulated two hypothetical groups with differential reproductive success and estimated the detection power of this difference based on the demographics of Burrishoole salmon (i.e. census size and reproductive success of the cohort; Figure 2 and Table 1) and the successfully genotyped proportions of the parental and offspring cohorts (Table 1). For Burrishoole salmon, such groups could represent several life history traits with an apparent (i.e. observable) dichotomy in the population, such as the duration of the ocean migration (1 year sea-winter vs. multiyear sea-winter), early maturation in males (mature male parr vs. sea-migrating males), or origin of stock (wild vs. hatchery). For the analysis, we first arbitrarily categorised the parental individuals within a cohort into one of the two ontological groups that differ in reproductive success (males and females separately). We define reproductive success of an individual as the number of offspring of an individual that return to reproduce four years later. Then, we modelled the reproductive success of every individual within the parental cohort using a negative binomial distribution, with dispersion parameters of 0.25 and 0.75 for males and females, respectively (Additional file 4).

The dispersion parameters within each sex were selected arbitrarily to reflect a realistic distribution of reproductive success in salmon, such that male reproductive success variance is higher than that of females (i.e., Bateman’s principle [61]), and more males than females fail to reproduce (Additional file 4). The reproductive success of parental individuals summed up to the numbers of offspring in the empirical Burrishoole cohorts (i.e. empirical reproductive success of the cohort; Table 1), reflects the reproductive success difference between the two groups. The ratio of the parental group with higher reproductive success within the cohort was set to be 0.01, 0.05, 0.15, 0.3, 0.5, 0.7, 0.85, 0.95 or 0.99. Next, the individuals in the offspring cohort were allocated to a parent pair based on the above conditions. Finally, the parental and offspring cohorts were sampled from the population pool based on the empirical Burrishoole sampling proportions (Figure 2), and the observed RRS between parental groups was inferred from the ratio of progeny in the offspring cohort using Fisher’s exact test. To estimate power, we simulated the above routine 1,000 times for all six parent-offspring cohort pairs. We also included in the model the parentage assignment success rate, by parameterising assignment rate of simulated genotypes for 14 and 28 loci (see Parentage analysis of simulated genotypes section above), and contrasted the power when using relatively low (i.e. 14) and high (i.e. 28) number of markers.

### Estimating the power to measure heritability of a continuous trait

We evaluated the power to detect the heritability of a continuous trait in two parent-offspring cohort pairs for which both panels of microsatellite markers (29 in total) had been genotyped (1977–1981, and 1979–1983), so that the pedigree structure was maximised (see Results for optimum marker number); we then compared the power to estimate heritability when using 14 vs. 29 markers. A continuous trait with heritability ranging from 0 to 1 with 0.05 increments was generated using the pedantics package 1.04 [62] based on the cohort pedigree generated using MASTERBAYES (130,000 iterations, 30,000 burn-ins and a thinning interval of 50, probability threshold; p = 0.95). The unsampled male and female numbers and error rates for the MASTERBAYES routine were the same as above. Then, phenotypic data were generated 100 times for every heritability value, using the pedigree information. Next, heritability was estimated from the simulated phenotypic data with an animal model using the PEDIGREEMM package 0.2-4 [63] after a slight modification as in [64]. Finally, the range of estimated heritabilities and the ratio of significant estimates (*p* < 0.05) were calculated for every expected heritability value, using the likelihood ratio test as described in the RLRSIM package 2.0-12 [65].

## Results

The mean genotyping success over 29 loci (Panel 1 and Panel 2 combined) was 88.0%, ranging from 75.8% to 94.3% per locus (Table 2, Additional file 2). Within the 10 year sampling period, genotyping success was significantly dependent on the age of the specimen (Table 1; adjusted *R*^*2*^ = 0.50, *F*_*(1,8)*_ = 10.11, *p* = 0.013). The mean allelic richness among cohorts was between 10.2 and 11.4, with locus-specific levels ranging from 3.3 to 26.0 (Table 2, Additional file 2). The mean observed heterozygosity within a marker was similar among cohorts (mean of coefficient of variation = 0.08, SD = 0.05), but spanned a large interval among markers (0.145 to 0.872). Similarly, the polymorphism information content (PIC) ranged between 0.15 and 0.90. The markers generally did not deviate from HWE; within the Panel 1 markers, at most only one cohort out of 10 deviated from HWE per marker, and no cohort was consistently out of HWE (Additional file 2). Likewise, 10 out of 15 Panel 2 markers were in HWE in all four cohorts, but two markers (Sleel53b, SSD30b) departed from HWE in two out of four cohorts. Similar to the Panel 1 markers, no cohorts showed consistent deviations from HWE across the Panel 2 markers (Additional file 2). The estimated null allele frequencies were generally low, ranging from 0.001 to 0.051 and from -0.016 to 0.095 for the Panel 1 and Panel 2 markers, respectively (Table 2, Additional file 2). Pairwise genetic differentiation between temporal samples was very low with Weir and Cockerham’s *θ*_*ST*_ ranging between 0.002 and 0.008, suggesting a high level of temporal stability among cohorts within the ten year sampling period (Additional file 5). Likewise, Jost’s D metric among loci was generally low such that only 6% of loci per cohort exceeded 0.1 (Additional file 5).

The average error rate among loci due to allelic dropout (E1) was estimated to be low (0.02 mean, 0.01 SD), and the error rate due to stochastic genotyping errors (E2) was even lower (0.004 mean, 0.001 SD). The posterior distribution of unsampled parents deviated from the prior estimates (Table 3). The posterior unsampled dam estimates were generally lower, while the posterior unsampled sire estimates were markedly larger than the prior estimate in all but one cohort, suggesting a higher number of male individuals contributing to the gene pool than expected based on census size estimates and the assumed precocious male parr contribution (Table 3).

**Table 3:** Prior and posterior estimates of number of unsampled parents

**Cohort year**	**Dam**		**Sire**	
	**Prior**	**Posterior (95% CI)**	**Prior**	**Posterior (95% CI)**
1977	260	167 (120–228)	250	966 (412–3922)
1978	228	47 (35–63)	202	1161 (215–19463)
1979	386	192 (146–252)	341	513 (349–809)
1980	296	158 (114–222)	279	369(180–1097)
1981	140	77 (58–99)	181	110 (79–161)
1982	150	184 (111–306)	180	517 (176–2357)

Prior unsampled population numbers are based on census population, sex ratio and precocious male parr contribution estimates (see the Methods section for details). Posterior numbers (median, 95% CI) are estimated within a Bayesian framework simultaneously with parentage using MASTERBAYES.

### Parentage assignment simulations

**Figure 4:**
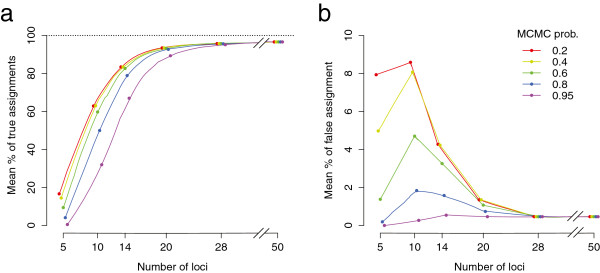
**Assignment success as a function of marker number. (a)** True assignment per cent. **(b)** False assignment per cent. Each data point is the mean of simulations of six parent-offspring cohort pairs, in which genotypes at each cohort pair were simulated 100 times using empirical allele frequency distribution. The points are jittered on the x axis to improve visual interpretation.

The parentage assignments with simulated datasets and different numbers of markers suggested that the optimal marker number for efficient parentage analysis in this system is approximately 20 to 28. Using 20 and 28 markers (with an average of 12 alleles per marker), a mean of 89.3% and 95.2%, respectively, of all parental links (as defined in the hypothetical pedigree) were identified at a conservative 95% threshold (Figure 4a, Additional file 6). In contrast, only 67.0% and 78.9% of parentage links were resolved using 14 markers at the 95% and 80% thresholds, respectively (Figure 4a, Additional file 6). Having more than 28 markers did not substantially increase the rate of correct parentage assignment. Having 50 markers resolved only 1.4% more parentage than 28 markers at the 95% threshold (Figure 4a, Additional file 6).

We tested five different probability thresholds for parentage assignment in the simulations. At all thresholds, the number of correctly and incorrectly assigned individuals was similar when using 20 or more markers. At lower numbers of markers, lower probability thresholds had higher number of correct links resolved, but at the same time, the number of incorrectly assigned individuals also increased (Figure 4b).

**Figure 5:**
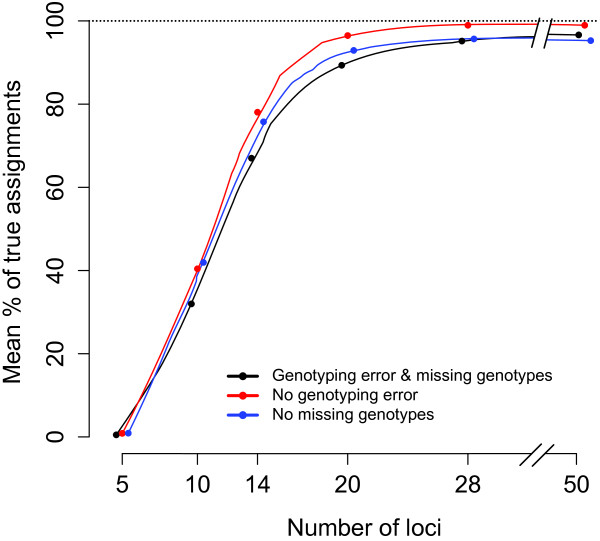
**Effect of missing genotypes and error rate on parentage assignment success.** Each data point is the mean of simulations of six parent-offspring cohort pairs, in which genotypes at each cohort pair were simulated 100 times using empirical allele frequency distributions.

Ignoring the genotyping error rate and missing genotype ratio while simulating genotypes resulted in overestimating the assignment power, such that 78.1% and 75.8% of individuals were assigned at 14 markers, respectively, while only 67.0% were assigned when these empirical error rates were included in the simulations (Figure 5, Additional file 6). The overestimation was marginal when 20 markers were used, such that 96.5% and 92.9% of individuals were assigned compared to 89.3% when the empirical error rates were included in the simulations.

### Empirical parentage assignments

Considering the six parent-offspring cohort pairs assessed with 14 microsatellites, a total of 341 pairs (297 and 44 female and male links, respectively) and 420 pairs (370 and 50 female and male parent-offspring links) were resolved at the 95% and 80% thresholds, respectively (Table 4, Additional file 7). Of these links, both parents were identified for 29 offspring, and 20 and 30 two-generational i.e. grandparent-parent-offspring links were detected at the 95% and 80% confidence thresholds, respectively (Table 4, Additional file 7). Overall, 502 (34%) and 609 (41%) of the 1,482 unique individuals included in the analysis were assigned some link in the pedigree, at the 95% and 80% thresholds, respectively. A total of 210 and 248 of the 901 potential parents (24% and 28% of individuals between 1977 and 1982) were identified at the 95% and 80% thresholds, respectively. Likewise, 312 and 391 of the 876 potential offspring (36% and 45% of individuals between 1981 and 1986) were identified at the 95% and 80% thresholds, respectively. There were two individuals who had spawned in consecutive years and had offspring identified (i.e., multiple spawners). The highest number of offspring assigned to a single dam was seven, compared to six assigned to a single sire (Additional file 7).

**Table 4:** Empirical pedigree statistics

	**‘77-‘81 (28 loci)**	**‘78-‘82**	**‘79-’83 (28 loci)**	**‘80-‘84**	**‘81-‘85**	**‘82-‘86**	**Across all cohorts**^**3**^
*Samples available*^*1*^							
Candidate mothers	120 (120)	61	193 (193)	111	135	129	749
Candidate fathers	22 (22)	6	74 (74)	13	17	20	152
Offspring	145 (151)	150	201 (205)	118	213	49	876
Total	287 (293)^2^	217	468 (472)^2^	242	365	198	1482
*Offspring with parent(s) identified*							
Maternal links identified	38 (51)	63	60 (93)	32	90	14	297
Paternal links identified	3 (3)	0	12 (21)	4	23	2	44
Total (at least one parent)	41 (54)	63	67 (109)	34	93	14	312
Total (both parents)	0 (0)	0	5 (5)	2	20	2	29
*Maternal half sib families*^4^							
Total family number	10 (11)	13	12 (18)	7	21	1	64
Mean family size	2.5 (2.8)	3.6	2.6 (2.9)	2.4	2.6	2.0	2.8
Maximum family size	4 (7)	6	6 (8)	3	5	2	7^5^
*Paternal half sib families*^4^							
Total family number	1 (1)	0	0 (4)	1	5	0	7
Mean family size	3 (3)	0	0 (2.5)	2	3.6	0	3.3
Maximum family size	3 (3)	0	0 (3)	2	6	0	6
*Other*							
Full-sib families^4^	0 (0)	0	0 (0)	0	4	0	4
Two generational links	-	-	-		-	-	20

Statistics are given for six parent – offspring cohorts and the combined pedigree using 14 microsatellite loci and 95% confidence threshold for all cohorts and combined pedigree, and also with 28 microsatellite markers for the two parent – offspring cohorts (i.e. ‘77 – ‘81 and ‘79 – ‘83, listed in parentheses).

^1^Successfully genotyped individuals that were included in the parentage analysis (see also: Table 1).

^2^The total number of individuals with 28 loci is slightly more than that of with 14 loci, as fewer individuals were filtered out due to the minimum number of loci genotyped (N <7) threshold.

^3^Based on 14 loci.

^4^Includes only families with more than 1 offspring identified.

^5^Maximum family size when cohorts’ combined is more than that of individual cohorts’ number due to a multiyear spawner having the highest clutch number.

Adding Panel 2 markers (29 markers in total) increased the overall parentage assignment success (Table 4, Additional file 7), such that 1.3 and 1.6 times more individuals were assigned to parent-offspring cohort pairs at the 95% threshold with 29 markers than with 14 markers in 1977–1981 and 1979–1983, respectively (Table 4, Additional file 7). These ratios are not significantly different from the simulated expectations (chi-square test; χ^2^ = 0.417, d.f. = 1, p = 0.52, and χ^2^ = 0.111, d.f. = 1, p = 0.74 for the 1977–1981 and 1979–1983 parent-offspring cohorts, respectively).

### Power to estimate relative reproductive success (RRS) among individuals

**Figure 6:**
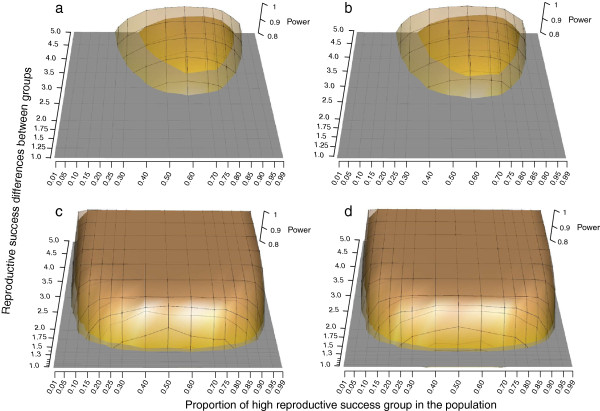
**Power to detect relative reproductive success.** Simulations are performed using demographic and sampling information of Burrishoole Atlantic salmon, and with varying group size proportions between high and low reproductive success groups. Panels a and b show average power within cohorts, for females **(a)**, and when both sexes combined **(b)**. Panels **c** and **d** show the power when cohorts’ information is combined for females **(c)**, and when both sexes combined **(d)**. Only higher than 80% power are shown. Tick marks on the xy plane and mesh vertices correspond to simulated points. Note that simulations are performed at finer intervals when a finer resolution is required, i.e. when comparing smaller reproductive success differences. The outer and the inner surfaces show results when parentages are resolved with 28 and 14 loci, respectively. (See Additional file 8 for full surface views, and male specific power curves).

Within a cohort, two to three fold differences in reproductive success between two groups (see Methods for details) could be detected in females (Figure 6a, Additional file 8), while the detection of even larger differences in reproductive success in males was unlikely (see Additional file 8). Combining individuals of both sexes somewhat improved the power compared to the female group only (Figure 6b). Detecting RRS had the highest power when the number of individuals in each group was balanced (r = 0.5, Figure 6, Additional file 8). Detecting the RRS between groups was more likely when the group with higher reproductive success was less common (r < 0.5, Figure 6). More subtle differences in reproductive success were detected when all cohort information was combined, such that differences in females as low as 1.5-fold were detected (Figure 6c), with a wide detection margin on the proportions of groups in the population. When cohorts were pooled, three to four times differences in male relative reproductive were resolved with about 50% power (Additional file 8). Using 28 markers compared to 14 improved the detection threshold such that close to a 1 fold gain in reproductive success detection was achieved across cohorts (Figure 6a, b). When cohort information was combined, improvement in detection was mostly limited to conditions when initial group size (i.e. parental) proportions were very unbalanced (i.e. when high reproductive success group comprise less than 15% of the total number, Figure 6c, d).

### Detecting power to estimate heritability of a continuous trait

**Figure 7:**
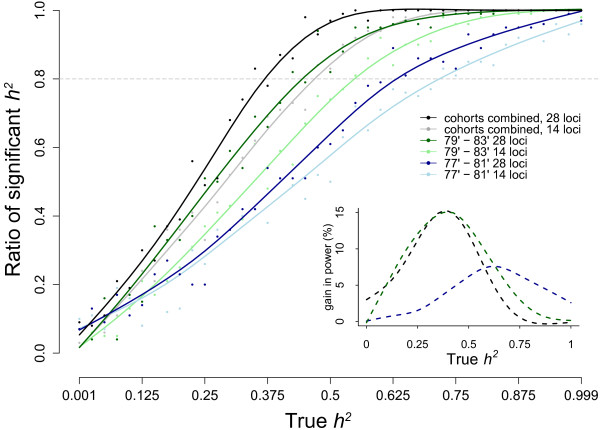
**Power to detect heritability of a continuous character in two Burrishoole Atlantic salmon cohorts.** Y axis shows proportions of significant heritability estimates at a given expected heritability value. Horizontal dashed line indicates 80% power for detecting heritability. Shades of blue, green and black are data for the parent-offspring cohort pairs for 1977–1981 and 1979–1983 years, and both dataset combined, respectively. Darker and lighter shades indicate pedigrees resolved at 29 and 14 loci, respectively. The inlet shows per cent power gain in power when pedigrees were resolved at 29 loci.

Using 29 loci, a continuous trait with a moderate heritability (i.e., 40-55%) can be detected with 80% or higher power (Figure 7). Standard deviations of heritabilities in simulations spanned a median range of 0.33 and 0.21 for the 1977–1981 and 1979–1983 cohort pairs, respectively (Additional file 9). The power to detect heritability was higher in the 1979–1983 cohort pair, which is likely the result of the higher number of parent-offspring links resolved in the parentage analysis (Table 4). When both cohorts were combined, the power of analysis improved so that approximately 30% heritability was detected with high power (Figure 7) and with lower standard deviations (i.e. median 0.17) (Additional file 9). Resolving the pedigree with 29 loci substantially improve the power compared to using 14 loci, and this was manifested more at low to moderate heritabilities with up to 15% power increase (See inset in Figure 7).

## Discussion

**Figure 8:**
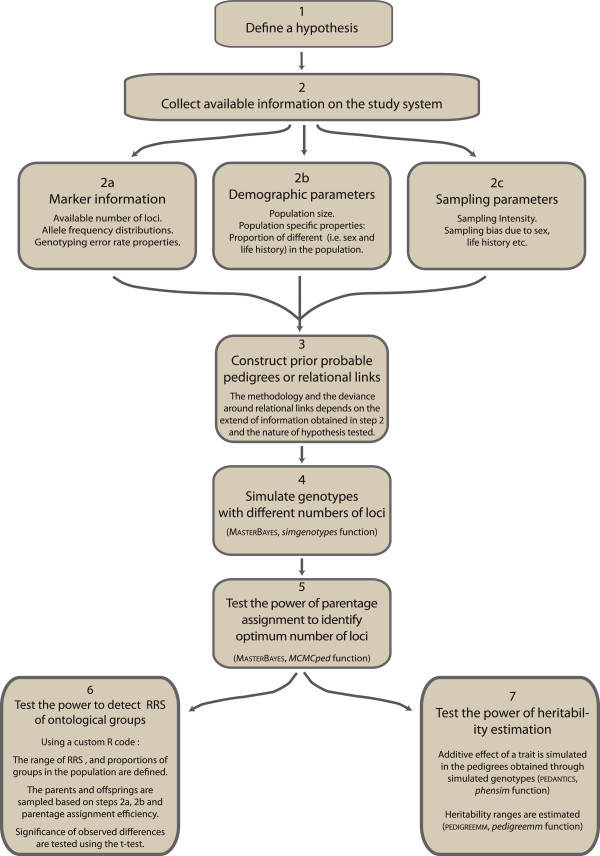
Flow chart depicting the steps to evaluate the feasibility of testing a hypothesis based on pedigree reconstruction using the available empirical information.

We have presented simulation-based approaches aimed at optimising the quantity and quality of pedigree information that can be obtained from a partially sampled wild population. Using simulations parameterised by empirical data from the Burrishoole Atlantic salmon, we have illustrated a robust procedure (summarised in Figure 8) for determining the optimum number of markers required to efficiently maximise the accuracy of parentage assignments, given the samples and molecular markers available in a system. Similarly, the accuracy and power of outputs of subsequent downstream analyses were quantified. On the basis of these evaluations, we conclude that the genetic information acquired on Burrishoole Atlantic salmon is suitable for detecting variation in reproductive success less than two fold between different groups within the cohorts, and also for estimating moderate heritability values for continuous life history traits.

Many studies have successfully identified parentage in wild fish populations with incomplete parental sampling using 15 or less microsatellite markers e.g. [27,35,57,66]. However, while explicit statistical methods are presented within parentage assignment packages to minimise false assignment rates at a low number of loci (i.e., type I errors) [1,67], generally, no empirical assessments are available to identify the proportion of non-assigned true parents in the parental pool (i.e., type II errors) [67]. Maximising the number of parental links that can be identified is crucial in achieving adequate power for testing the various hypotheses and questions that long-term population studies like this aspire to address, which in turn makes it important to maximise and evaluate the quality of parentage assignments, especially within the context of incomplete sampling. On the other hand, a trade-off exists between the occurrence of type I vs. type II errors. A strategy overlooking pedigree error, in the interest of maximising pedigree links, may not be optimal as pedigree errors may also be associated with low power in upstream analyses (i.e. estimation of heritability [16]). It is possible that general assumptions about parentage assignment efficiency can be demonstrated within a theoretical framework i.e. [59,67]; however, empirical approaches, as we have demonstrated in this study, provide system-specific parameters (i.e., population size, sampling ratio, genotyping error rate, missing genotype information, empirical marker characteristics) to be utilised in the simulations, and thus allows for the use the minimum number of loci for parentage assignment analysis without excluding true parents, to be determined (see Figure 8). In the Burrishoole system, we concluded that genotyping with at least 20 (and preferably 28) markers (i) maximised parentage assignments without leaving substantial numbers of true parents unassigned (Figure 4); and (ii) improved the biological insights in both RRS and heritability analyses. Therefore, we highly recommend an evaluation of the power of a specific study by explicitly modelling the properties of the DNA markers used in the study as well as the demographical parameters.

Knowledge of the numbers of unsampled individuals is critical for efficient parentage assignments in the likelihood-based categorical allocation approaches (e.g. in cervus[50] and MASTERBAYES[56]), such that the probability of genotypes to be present among unsampled parents is included in confidence interval estimations of assigned parents [57,59]. High error rates (i.e., incorrect assignments) are expected in likelihood-based parentage analyses (e.g. cervus) if the number of unsampled parents is incorrectly estimated in the model [57]. Although estimating unsampled breeders is relatively easy in closed populations or well-established model systems, it is challenging in partially sampled populations and particularly problematic in salmon, where mature male parr may contribute substantially to the breeding pool. Furthermore, in the case of the Burrishoole system, hatchery origin individuals might also potentially contribute to the spawning population in the wild. Therefore, the simultaneous estimation of unsampled parents and parent-offspring linkages within a Bayesian framework is preferable for the Burrishoole system and for other systems where there is some doubt as to the number of unsampled potential parents. Indeed, in the Burrishoole system, the unsampled male number greatly exceeds the total enumerated number of returning males that have migrated to sea, which suggests a significant parr contribution to the breeding pool (Table 3) that may exceed the previous estimates of 30% of the total breeding pool [45] (but also see [34] for a review). However, posterior distribution based estimates of unsampled females are lower than the prior estimates in five out of six parent cohorts, which is expected, as not all unsampled females in the system are expected to breed or to have sampled offspring.

An important aspect of parentage analysis in the wild is that it enables comparisons of the reproductive success between different groups of fish to be undertaken e.g. [19,59,68,69]. Groups of individuals with recorded differences in life histories (e.g. sea-age of maturity, male parr maturation) as well as salmon with different wild and hatchery provenance can be compared using family reconstructions. The latter has been of particular interest in this system due to its potential impact on the genetic makeup and productivity of the Burrishoole wild recipient population [25,38,41]. We showed using population parameters based on the Burrishoole system that differences in reproductive success of 2.5 fold can be detected successfully between different groups of fish (Figure 6a and b, Additional file 8), while 1.5 fold differences can be detected when cohorts are combined (Figure 6c and d, Additional file 8). These differences are much smaller than, for example, the empirical estimation of marine mortality rates between hatchery origin and wild individuals from the Burrishoole system (i.e., ranched individuals are five times less likely to survive [38]), though more recent estimates, based on the five year average from 2007 to 2011, show that Burrishoole hatchery origin fish were only two times less likely to survive [70]. Unlike females, however, the low proportion of males sampled results in no power to detect the RRS among males in different groups (Additional file 8). We suggest that estimating the RRS among wild-born fish that may have been descended from hatchery or wild-origin fish might be one of the most tractable applications of the pedigree reconstruction approach in the Burrishoole system. Using 28 loci rather than just 14 improved the power in RRS estimation substantially (Figure 6a, b), which suggests that in the Burrishoole system, a difference in reproductive success within a cohort is more likely to be captured when genotyping is performed with 28 markers. This gain in power is clearer only when group proportions are overly skewed in the population (i.e. proportion of high reproductive success group <0.15, see Figure 6c, d). This scenario is quite likely, given that many dichotomies in life history or phenotype may be present with skewed proportions, such as sea-winterism or ranched proportions in the Burrishoole system [25,42,44].

Among Burrishoole salmon in this study, only moderate heritability values for a continuous trait can be detected with sufficient power. The values obtained are comparable to the heritability estimates of life history and morphological traits for salmonids, most of which have been reported under artificial environmental settings (for a review, see: [28]). The standard deviations of the simulated heritability estimates of this study (i.e., 0.23 and 0.16 for 1977–1981 and 1979–1983 parent-offspring cohorts, respectively, and 0.14 for both cohorts combined) are well within the range of distribution of standard errors reported in [28], where the mean of the standard errors of 2,108 heritability estimates is 0.15. At present, there are only a handful of heritability estimates for salmonids in the wild e.g. [17,31-33], illustrating the value of being able to accurately estimate trait heritability in this and other systems. Using 29 loci instead of 14 improved the detection power markedly, especially in the heritability ranges where most of the salmonid values were observed (see inset in Figure 7, [28]). Furthermore, the estimates become more precise with higher number of loci (Additional file 9); when using 29 loci, the power increase in 79′-83′ parent offspring cohort is as much as the power gained when the two parent-offspring cohort information is combined, but using 14 loci (Figure 7). When using cohort information separately in heritability estimates it is important to measure evolutionary dynamics over time, especially upon changing environmental conditions [7,71,72], or when phenotypic variation is introduced by means of supplementation.

Another factor that should be considered is the potential influence of maternal effects on the accuracy of heritability estimates, particularly given that the Burrishoole pedigree is currently heavily reliant on maternal connections (Figures 2 and 4). This situation would have been problematic if the traits of interest were measured in young fish. However, as the maternal effect (environmental) in salmonids rarely persists into maturity (i.e., >9 months) [73]; it is likely that only a negligible portion of the estimated variance component can be attributed to the phenotype of the mother.

## Conclusions

This study demonstrates the feasibility of pedigree-based research to estimate key population parameters, which in turn can be used to resolve difficult ecological and evolutionary questions in wild populations. The approaches described above provide a robust methodological framework for evaluating historically sampled populations for pedigree-based research and to retrieve valuable quantitative genetic information, even where sampling represents only a proportion of the total census population.

## Competing interests

The authors declare that they have no competing interests.

## Authors’ contributions

PMG, CRP, SEJ, TA, TFC, PAP conceived and designed the study. RP, DC, GR, PMG oversaw sample collection and archiving. TA, SEJ, CRP designed the simulations and analysed the data. TA, CRP, PMG, TR wrote the article. All authors commented on and approved the final manuscript.

## Authors’ information

Philip McGinnity and Craig R Primmer shared senior authorship.

## Supplementary Material

Additional file 1 Primers for each marker used in the study. Click here for file

Additional file 2 Descriptive statistics of markers used in the study. Click here for file

Additional file 3 Error rate estimation procedure. Click here for file

Additional file 4 Assumed lifetime reproductive success variation of Burrishoole Atlantic salmon for females (a), and males (b). Click here for file

Additional file 5 Differentiation metrics (CI) between parental and offspring cohorts of microsatellite markers used in the study. Click here for file

Additional file 6 Assignment success in simulations (medians and standard deviation). Click here for file

Additional file 7 Additional pedigree statistics and figures. Click here for file

Additional file 8 Power to detect relative reproductive success among groups of fish based on Burrishoole Atlantic salmon demography and sampling information, additional figures. Click here for file

Additional file 9 Ranges of heritability estimates for a given heritability value. Click here for file
