# Supplementary material for: Molecular pedigree reconstruction and estimation of evolutionary parameters in a wild Atlantic salmon river system with incomplete sampling: a power analysis
Source: BMC Evol Biol. 2014 Mar 31;14:68. doi: 10.1186/1471-2148-14-68 (PMC4021076; doi:10.1186/1471-2148-14-68)
Supplement: Additional file 1 — Primers for each marker used in the study. [file 1471-2148-14-68-S1.docx]

**Additional File 1: Primers for each marker used in the study.** All markers except Sssp3016 (Gene bank accession number: AY372820) are listed in [46]**.** Fluorescent dye, working primer concentration, multiplex group and genotyping groups (panel) of each primer pair are indicated in the table ^1^.

| Locus | Label | Panel^2^ | Multiplex number | Primer (µM) |
| --- | --- | --- | --- | --- |
| CA060177 | pet | 1 | MP1 | 0.36 |
| Ssa 407 | ned | 1 | MP1 | 0.36 |
| CA064557 | ned | 1 | MP1 | 0.27 |
| Ssa197 | vic | 1 | MP1 | 0.10 |
| Ssa 171 | fam | 1 | MP1 | 0.35 |
| Ssa 412 | vic | 1 | MP1 | 0.27 |
| CA040282 | pet | 1 | MP2 | 0.10 |
| SSsp2216 | fam | 1 | MP2 | 0.20 |
| CA039983 | pet | 1 | MP2 | 0.10 |
| SSsp2215 | fam | 1 | MP2 | 0.10 |
| CA047220 | vic | 1 | MP2 | 0.30 |
| SSsp2210 | ned | 1 | MP2 | 0.30 |
| Ssa85 | vic | 1 | MP2 | 0.20 |
| CA062844 | pet | 1 | MP2 | 0.10 |
| Ssleer 15.1 | fam | 2 | MP3 | 0.36 |
| SSsp1605 | vic | 2 | MP3 | 0.04 |
| Ssa124 | vic | 2 | MP3 | 0.20 |
| Ssa202 | ned | 2 | MP3 | 0.40 |
| Ssf43 | ned | 2 | MP3 | 0.10 |
| Ssosl 438 | fam | 2 | MP3 | 0.25 |
| Sssp3016 | pet | 2 | MP3 | 0.30 |
| Ssd30 | vic | 2 | MP4 | 0.40 |
| Ssosl25 | ned | 2 | MP4 | 0.30 |
| Sleen82 | fam | 2 | MP4 | 0.20 |
| Ssa98 | ned | 2 | MP4 | 0.20 |
| Sleei53 | fam | 2 | MP4 | 0.15 |
| Sssp2201 | fam | 2 | MP4 | 0.40 |
| EST405 | ned | 2 | MP4 | 0.33 |
| Ssosl311 | vic | 2 | MP4 | 0.40 |

^1^PCR reactions were performed at 8 µl consisted 1 µl of genomic DNA (variable concentration), 0.04 to 0.40 µM of each primer, and 1X QIAGEN multiplex PCR master mix (Qiagen Inc. Valencia, CA, USA). The PCR program was as follows: 15 minutes at 95 ˚C for initial denaturing followed by 36 cycles of denaturing at 94 ˚C for 30 seconds, annealing at 60/59 ˚C for 1.5 minutes and elongating at 72 ˚C for 1 minute with final elongation at 72 ˚C for 5 minutes. Annealing temperature was 59 for MP1 and 60 for the remaining multiplex reactions.

^2^The multiplex PCRs were pooled for fragment analysis as follows: for panel 1, 1.6 µl of MP1 and 1.7 µl of MP2 were pooled to 100 µl of H_2_O and for panel 2, 1.5µl of MP3 and 1.5 µl of MP4 were pooled to 100 µl of H_2_O.
