# Supplementary material for: Molecular pedigree reconstruction and estimation of evolutionary parameters in a wild Atlantic salmon river system with incomplete sampling: a power analysis
Source: BMC Evol Biol. 2014 Mar 31;14:68. doi: 10.1186/1471-2148-14-68 (PMC4021076; doi:10.1186/1471-2148-14-68)
Supplement: Additional file 2 — Descriptive statistics of markers used in the study. [file 1471-2148-14-68-S2.docx]

**Additional file 2. Descriptive statistics of markers used in the study.** Panel 1 markers (N= 14) were used for all analyses and for all cohorts (total number of individuals = 1640), while Panel 2 markers (N=15, number of individuals =832) were used only for a subset of cohorts (i.e., 1977, 1979, 1981, 1983) in the heritability power estimation and to verify the simulation results (see the Materials and Methods section for details). Ho= observed heterozygosity, Ho= expected heterozygosity, Sig. HWE: number of cohorts that significantly deviate from Hardy-Weinberg equilibrium, PIC: polymorphism information content. Values in parenthesis show the ranges among marked cohorts.

| Marker name | Mean number of genotyped individuals | % Mean genotyping success | Allele range | Mean allele number | Mean allelic richness | | Mean *H_O_* | | Mean *H_E_* | | Null allele frequency | Mean exclusion probability | Mean PIC | Sig. HWE |  |
| --- | --- | --- | --- | --- | --- | --- | --- | --- | --- | --- | --- | --- | --- | --- | --- |
| Panel 1 (N= 10 cohorts) | |  |  |  |  |  | |  | |  | |  |  |  | |
| SSsp2215 | 150 (52-271) | 93.7 | 105-174 | 15.4 (14-17) | 13.2 (12.1-15.2) | 0.872 (0.83-0.907) | | 0.884 (0.87-0.893) | | 0.006 (-0.009-0.027) | | 0.68 (0.65-0.70) | 0.87 | 0 | |
| Ssa171 | 147 (52-261) | 91.4 | 202-243 | 11.3 (9-13) | 9.5 (8.6-11.0) | 0.772 (0.657-0.837) | | 0.808 (0.791-0.831) | | 0.023 (-0.020-0.091) | | 0.51 (0.47-0.55) | 0.79 | 0 | |
| SSsp2216 | 144 (52-256) | 88.7 | 272-337 | 16.2 (15-18) | 14.9 (14.0-15.8) | 0.845 (0.655-0.91) | | 0.906 (0.897-0.912) | | 0.036 (0.001-0.159) | | 0.73 (0.71-0.75) | 0.9 | 0 | |
| Ssa85 | 135 (48-214) | 83.8 | 115-150 | 15.6 (13-19) | 12.8 (11.4-14.3) | 0.849 (0.799-0.917) | | 0.850 (0.826-0.869) | | 0.001 (-0.028-0.023) | | 0.60 (0.54-0.65) | 0.84 | 0 | |
| Ssa197 | 147 (52-256) | 91.6 | 159-267 | 19.0 (17-22) | 15.5 (13.4-17) | 0.838 (0.77-0.902) | | 0.863 (0.836-0.884) | | 0.015 (-0.022-0.062) | | 0.63 (0.57-0.67) | 0.85 | 0 | |
| Ssa412 | 148 (52-265) | 91.7 | 289-301 | 4.1 (3-5) | 3.3 (2.9-4.4) | 0.399 (0.313-0.498) | | 0.416 (0.351-0.48) | | 0.021 (-0.050-0.085) | | 0.07 (0.05-0.09) | 0.34 | 1 | |
| EST107 | 132 (47-228) | 80.7 | 256-367 | 6.4 (5-9) | 5.9 (5.0-6.6) | 0.571 (0.383-0.712) | | 0.625 (0.525-0.693) | | 0.051 (-0.017-0.237) | | 0.24 (0.15-0.31) | 0.6 | 1 | |
| SSsp2210 | 149 (52-271) | 92.9 | 112-160 | 10.9 (9-12) | 9.7 (8.5-10.8) | 0.705 (0.664-0.75) | | 0.712 (0.67-0.775) | | 0.004 (-0.048-0.048) | | 0.34 (0.28-0.42) | 0.67 | 0 | |
| Ssa407 | 140 (48-254) | 86.3 | 117-311 | 17.4 (12-22) | 14.0 (11.3-15.4) | 0.796 (0.716-0.85) | | 0.826 (0.781-0.853) | | 0.019 (-0.006-0.068) | | 0.55 (0.45-0.60) | 0.81 | 1 | |
| EST28 | 133 (49-236) | 81.7 | 335-375 | 4.4 (3-5) | 3.9 (3.0-5.0) | 0.210 (0.145-0.25) | | 0.221 (0.156-0.287) | | 0.025 (-0.055-0.078) | | 0.02 (0.01-0.04) | 0.21 | 1 | |
| EST41 | 148 (52-262) | 92.9 | 123-180 | 16.1 (13-20) | 12.7 (10.9-14.6) | 0.785 (0.743-0.831) | | 0.833 (0.823-0.857) | | 0.029 (-0.005-0.054) | | 0.56 (0.54-0.61) | 0.82 | 0 | |
| EST68 | 146 (52-262) | 89.9 | 186-198 | 6.3 (5-7) | 5.9 (5.0-7.0) | 0.613 (0.519-0.648) | | 0.634 (0.603-0.671) | | 0.019 (-0.030-0.112) | | 0.23 (0.19-0.27) | 0.57 | 0 | |
| EST123 | 146 (52-262) | 89.5 | 269-279 | 5.9 (5-7) | 5.5 (5.0-6.0) | 0.681 (0.557-0.808) | | 0.715 (0.657-0.743) | | 0.024 (-0.047-0.103) | | 0.34 (0.26-0.38) | 0.68 | 0 | |
| EST19 | 145 (52-260) | 89.7 | 284-369 | 19.5 (16-24) | 16.2 (14.1-18.4) | 0.828 (0.741-0.9) | | 0.869 (0.852-0.888) | | 0.026 (-0.015-0.076) | | 0.64 (0.60-0.68) | 0.86 | 0 | |
| Panel 1 mean. | 143.6 | 88.9 |  | 12.0 | 10.2 | 0.697 | | 0.725 | | 0.021 | | 0.44 | 0.70 | 0.29 | |
|  |  |  |  |  |  |  | |  | |  | |  |  |  | |
| Panel 2 (N= 4 cohorts) | |  |  |  |  |  | |  | |  | |  |  |  | |
| Ssosl438 | 181 (144-229) | 87.1 | 112-170 | 11.5 (11-13) | 11.0 (10.0-12.9) | 0.799 (0.769-0.833) | | 0.801 (0.787-0.828) | | -0.001 (-0.019-0.011) | | 0.49 (0.47-0.55) | 0.77 | 1 | |
| Sleel53 | 196 (143-275) | 94.4 | 166-189 | 5.5 (4-7) | 5.1 (4.0-6.3) | 0.476 (0.413-0.503) | | 0.504 (0.419-0.538) | | 0.023 (-0.007-0.038) | | 0.13 (0.08-0.16) | 0.48 | 2 | |
| Ssleer151 | 193 (141-270) | 92.7 | 185-189 | 3.8 (3-4) | 3.6 (3.0-4.0) | 0.510 (0.47-0.576) | | 0.502 (0.494-0.506) | | -0.008 (-0.068-0.023) | | 0.10 (0.10-0.11) | 0.39 | 0 | |
| Sleen82 | 191 (137-261) | 91.9 | 206-226 | 8.2 (8-9) | 7.8 (7.2-8.2) | 0.642 (0.559-0.678) | | 0.659 (0.614-0.687) | | 0.016 (-0.008-0.057) | | 0.27 (0.22-0.30) | 0.61 | 0 | |
| SSsp2201 | 174 (127-234) | 83.5 | 249-333 | 25.5 (24-27) | 24.7 (24.0-25.4) | 0.871 (0.772-0.917) | | 0.899 (0.888-0.913) | | 0.017 (-0.002-0.066) | | 0.72 (0.69-0.75) | 0.9 | 0 | |
| Ssosl311 | 185 (138-256) | 88.8 | 118-214 | 22.0 (18-25) | 20.7 (18.0-22.7) | 0.852 (0.82-0.903) | | 0.880 (0.871-0.886) | | 0.017 (-0.009-0.035) | | 0.67 (0.65-0.68) | 0.88 | 0 | |
| Ssa124 | 191 (136-262) | 91.8 | 172-222 | 9.2 (8-10) | 8.5 (8.0-9.0) | 0.640 (0.588-0.689) | | 0.660 (0.635-0.685) | | 0.015 (-0.008-0.041) | | 0.27 (0.25-0.30) | 0.62 | 0 | |
| SSD30 | 188 (140-259) | 90.4 | 237-292 | 4.8 (4-6) | 4.6 (3.7-5.6) | 0.145 (0.106-0.171) | | 0.150 (0.102-0.172) | | 0.018 (-0.018-0.093) | | 0.01 (0.00-0.01) | 0.15 | 2 | |
| SSsp1605 | 178 (126-236) | 85.8 | 120-292 | 10.2 (7-12) | 9.6 (7.0-10.8) | 0.755 (0.698-0.821) | | 0.788 (0.757-0.798) | | 0.021 (-0.014-0.041) | | 0.47 (0.41-0.49) | 0.76 | 0 | |
| SSf43 | 191 (142-253) | 91.9 | 103-120 | 7.2 (6-8) | 6.9 (5.5-8.0) | 0.634 (0.605-0.682) | | 0.645 (0.632-0.664) | | 0.007 (-0.039-0.053) | | 0.24 (0.22-0.26) | 0.58 | 1 | |
| Ssosl25 | 184 (138-249) | 88.2 | 139-172 | 11.2 (10-12) | 11.0 (9.9-12.0) | 0.786 (0.757-0.819) | | 0.759 (0.752-0.776) | | -0.016 (-0.031-0.006) | | 0.42 (0.41-0.45) | 0.74 | 0 | |
| Ssa98 | 168 (116-224) | 80.9 | 184-228 | 8.2 (8-9) | 8.0 (7.5-9.0) | 0.493 (0.375-0.621) | | 0.586 (0.562-0.641) | | 0.095 (0.020-0.208) | | 0.20 (0.18-0.25) | 0.55 | 1 | |
| Ssa202 | 167 (120-215) | 80.2 | 231-268 | 11.0 (10-12) | 10.5 (9.7-12.0) | 0.733 (0.675-0.773) | | 0.784 (0.758-0.795) | | 0.035 (0.014-0.082) | | 0.46 (0.41-0.48) | 0.76 | 0 | |
| EST405 | 158 (110-207) | 75.8 | 96-387 | 27.2 (26-29) | 26.0 (24.6-28.1) | 0.871 (0.809-0.944) | | 0.906 (0.899-0.917) | | 0.020 (-0.016-0.054) | | 0.74 (0.72-0.77) | 0.9 | 0 | |
| Sssp3016 | 176 (118-251) | 84.4 | 91-252 | 13.5 (12-16) | 13.1 (12.1-15.6) | 0.713 (0.649-0.79) | | 0.741 (0.697-0.795) | | 0.018 (-0.005-0.036) | | 0.39 (0.32-0.48) | 0.71 | 0 | |
| Panel 2 mean. | 181.4 | 87.2 |  | 11.9 | 11.4 | 0.661 | | 0.684 | | 0.018 | | 0.37 | 0.65 | 0.46 | |
