# Supplementary material for: Molecular pedigree reconstruction and estimation of evolutionary parameters in a wild Atlantic salmon river system with incomplete sampling: a power analysis
Source: BMC Evol Biol. 2014 Mar 31;14:68. doi: 10.1186/1471-2148-14-68 (PMC4021076; doi:10.1186/1471-2148-14-68)
Supplement: Additional file 3 — Error rate estimation procedure. [file 1471-2148-14-68-S3.docx]

**Additional file 3: Error rate estimation procedure.**

Error rate estimation was carried out over time and separately for each locus since both factors had a significant effect on error rate (see, table and figure below).

Because the total number of informative individuals (i.e. replicate genotypes) was not heterogenous among cohorts, and that some replicates were not in the same cohort year (i.e. individuals that were sampled in two consecutive years. see text for details), we adopted a sliding window analysis to enrich the number of replicates within a time point. The error rate was estimated by pooling individuals from three subsequent years and the window was iterated yearly for the next three years. Then for a particular cohort, error rate was averaged (mean) over the sliding windows that the cohort is an element of.

**Table additional file 3: Effect of time and locus to error rate.**

|  | factor | df | mean sq | sum sq | F value | p value |
| --- | --- | --- | --- | --- | --- | --- |
| E1 (allelic dropout) | time | 13 | 0.00794 | 0.000611 | 19.1 | <0.001 |
|  | locus | 1 | 0.010108 | 0.010108 | 316.1 | <0.001 |
|  | time x locus | 13 | 0.004183 | 0.000322 | 10.1 | <0.001 |
| E2 (stochastic error rate) | time | 13 | 0.00023 | 1.77E-05 | 11.2 | <0.001 |
|  | locus | 1 | 1.25E-07 | 1.25E-07 | 0.07 | 0.77 |
|  | time x locus | 13 | 7.66E-05 | 5.90E-06 | 3.7 | <0.001 |

**Figure additional file 3: Effect of time and locus to error rate. Each color shows a different locus.**
