# Supplementary material for: Molecular pedigree reconstruction and estimation of evolutionary parameters in a wild Atlantic salmon river system with incomplete sampling: a power analysis
Source: BMC Evol Biol. 2014 Mar 31;14:68. doi: 10.1186/1471-2148-14-68 (PMC4021076; doi:10.1186/1471-2148-14-68)
Supplement: Additional file 4 — Assumed lifetime reproductive success variation of Burrishoole Atlantic salmon for females (a), and males (b). [file 1471-2148-14-68-S4.pdf]

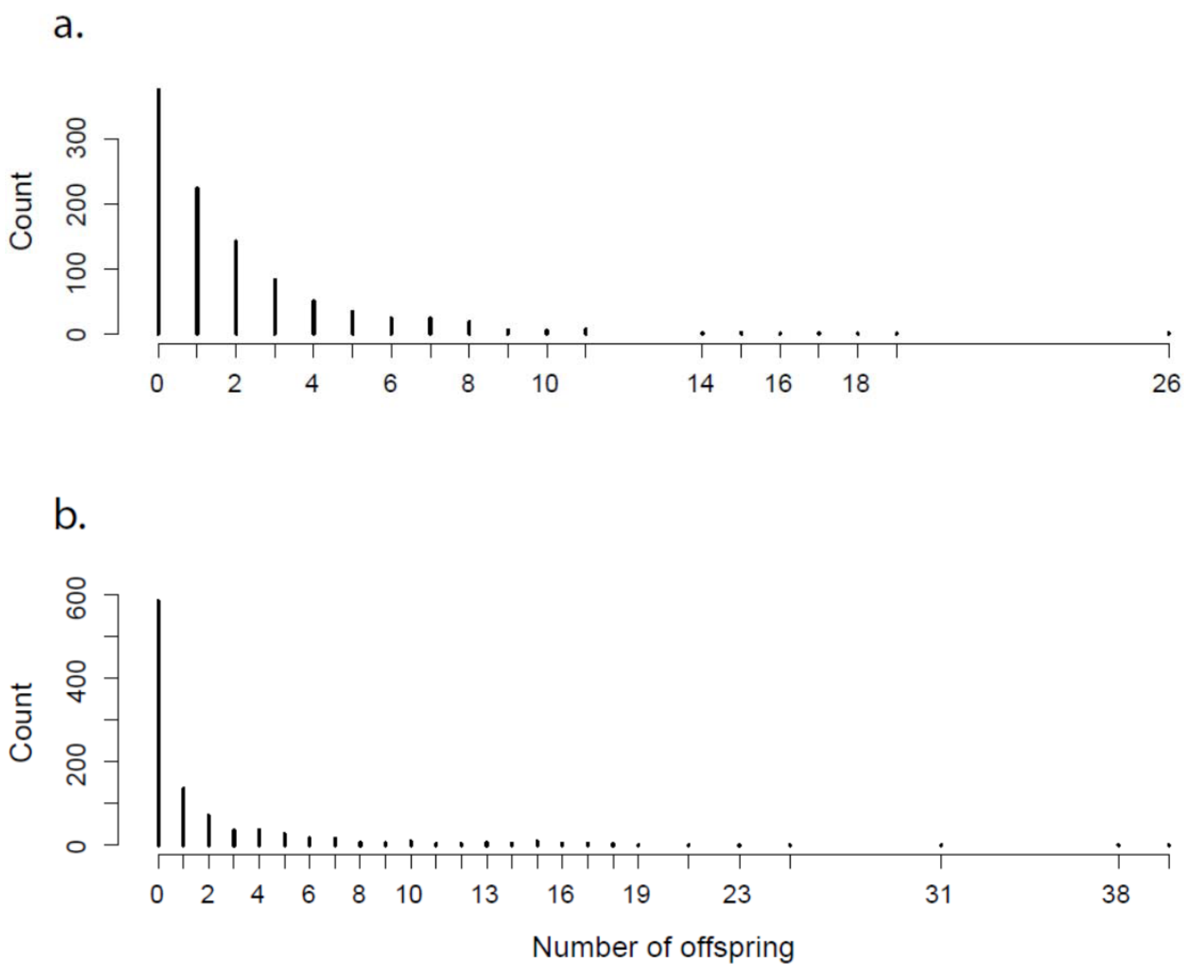

Additional file 4. Assumed lifetime reproductive success of Burrishoole Atlantic salmon for females (a), and males (b). The distributions are negative binomial distributions with dispersion parameters 0.25 and 0.75 for males and females, respectively. In the above example, the average reproductive success for each gender is assumed to be two (i.e. stable population size), while in the simulations average reproductive success is adjusted for empirical census population size (Table 1).
