# Supplementary material for: Molecular pedigree reconstruction and estimation of evolutionary parameters in a wild Atlantic salmon river system with incomplete sampling: a power analysis
Source: BMC Evol Biol. 2014 Mar 31;14:68. doi: 10.1186/1471-2148-14-68 (PMC4021076; doi:10.1186/1471-2148-14-68)
Supplement: Additional file 5 — Differentiation metrics (CI) between parental and offspring cohorts of microsatellite markers used in the study. [file 1471-2148-14-68-S5.docx]

**Additional file 5. Differentiation metrics (CI) between parental and offspring cohorts of microsatellite markers used in the study.**

|  |  | Parent offspring cohort pair | | | | | |
| --- | --- | --- | --- | --- | --- | --- | --- |
|  | Locus name | 77'-81' | 78'-82' | 79'-83' | 80'-84' | 81'-85' | 82'-86' |
| Jost’s *D* | SSsp2215 | 0.066(0.025-0.123) | 0.064(0.021-0.133) | 0.024(0.009-0.049) | 0.044(0.014-0.095) | 0.042(0.016-0.079) | 0.114(0.048-0.208) |
|  | Ssa171 | 0.020(0.005-0.049) | 0.047(0.014-0.103) | 0.018(0.004-0.042) | 0.042(0.009-0.100) | 0.020(0.005-0.048) | 0.065(0.021-0.136) |
|  | SSsp2216 | 0.090(0.038-0.160) | 0.118(0.052-0.209) | 0.054(0.021-0.104) | 0.060(0.024-0.113) | 0.042(0.015-0.084) | 0.127(0.058-0.218) |
|  | Ssa85 | 0.065(0.028-0.117) | 0.061(0.023-0.120) | 0.037(0.013-0.078) | 0.055(0.024-0.096) | 0.041(0.017-0.076) | 0.083(0.027-0.167) |
|  | Ssa197 | 0.040(0.014-0.082) | 0.062(0.022-0.136) | 0.037(0.015-0.068) | 0.049(0.015-0.108) | 0.037(0.011-0.082) | 0.089(0.036-0.179) |
|  | Ssa412 | 0.005(0.000-0.020) | 0.006(0.000-0.030) | 0.001(0.000-0.006) | 0.003(0.000-0.013) | 0.003(0.000-0.012) | 0.006(0.000-0.029) |
|  | EST107 | 0.023(0.003-0.059) | 0.026(0.004-0.070) | 0.006(0.001-0.020) | 0.018(0.004-0.042) | 0.012(0.001-0.035) | 0.029(0.004-0.080) |
|  | SSsp2210 | 0.019(0.005-0.049) | 0.027(0.006-0.072) | 0.009(0.002-0.025) | 0.014(0.004-0.033) | 0.016(0.003-0.045) | 0.027(0.007-0.066) |
|  | Ssa407 | 0.039(0.016-0.078) | 0.056(0.021-0.119) | 0.020(0.007-0.044) | 0.030(0.011-0.067) | 0.021(0.007-0.047) | 0.065(0.023-0.136) |
|  | EST28 | 0.002(0.000-0.009) | 0.002(0.000-0.009) | 0.001(0.000-0.003) | 0.002(0.000-0.007) | 0.001(0.000-0.005) | 0.010(0.001-0.031) |
|  | EST41 | 0.041(0.015-0.084) | 0.059(0.017-0.134) | 0.028(0.010-0.056) | 0.054(0.015-0.114) | 0.031(0.011-0.064) | 0.118(0.042-0.222) |
|  | EST68 | 0.011(0.002-0.035) | 0.019(0.002-0.064) | 0.006(0.001-0.019) | 0.008(0.001-0.029) | 0.008(0.001-0.028) | 0.021(0.003-0.069) |
|  | EST123 | 0.020(0.003-0.053) | 0.026(0.003-0.071) | 0.014(0.003-0.034) | 0.020(0.002-0.055) | 0.023(0.005-0.051) | 0.022(0.003-0.066) |
|  | EST19 | 0.053(0.022-0.102) | 0.072(0.027-0.147) | 0.033(0.013-0.064) | 0.048(0.016-0.102) | 0.038(0.014-0.078) | 0.110(0.047-0.205) |
|  | Global (N= 14) | 0.022(0.016-0.030) | 0.029(0.020-0.041) | 0.012(0.009-0.016) | 0.021(0.015-0.028) | 0.015(0.011-0.021) | 0.041(0.030-0.054) |
| Weir and Cockerham’s *Ɵ_ST_* | Global (N= 14) | 0.004(0.002-0.007) | 0.005(0.001-0.009) | 0.002(0.001-0.004) | 0.004(0.002-0.007) | 0.003(0.001-0.005) | 0.008(0.004-0.013) |

**Additional file 5. Table 1: Differentiation metrics (CI) between parental and offspring cohorts for panel 1 microsatellites.**

**Additional file 5.Table 2: Differentiation metrics (CI) between parental and offspring cohorts for panel 2 microsatellites.**

| Panel 2 |  | Parent offspring cohort pair | | |
| --- | --- | --- | --- | --- |
|  | Locus name | 77'-81' | 79'-81' |  |
| Jost’s *D* | Ssosl438 | 0.035(0.010-0.076) | 0.014(0.003-0.034) |  |
|  | Sleel53 | 0.020(0.004-0.046) | 0.005(0.001-0.014) |  |
|  | Ssleer151 | 0.004(0.000-0.016) | 0.003(0.000-0.013) |  |
|  | Sleen82 | 0.016(0.003-0.041) | 0.008(0.001-0.022) |  |
|  | SSsp2201 | 0.053(0.025-0.096) | 0.045(0.020-0.084) |  |
|  | Ssosl311 | 0.083(0.033-0.154) | 0.053(0.025-0.093) |  |
|  | Ssa124 | 0.015(0.002-0.042) | 0.015(0.002-0.038) |  |
|  | SSD30 | 0.002(0.000-0.006) | 0.001(0.000-0.002) |  |
|  | SSsp1605 | 0.044(0.011-0.100) | 0.020(0.004-0.051) |  |
|  | SSf43 | 0.008(0.001-0.026) | 0.012(0.001-0.035) |  |
|  | Ssosl25 | 0.017(0.004-0.040) | 0.012(0.003-0.029) |  |
|  | Ssa98 | 0.064(0.025-0.120) | 0.007(0.001-0.021) |  |
|  | Ssa202 | 0.026(0.007-0.060) | 0.021(0.004-0.051) |  |
|  | EST405 | 0.089(0.044-0.156) | 0.060(0.031-0.101) |  |
|  | Sssp3016 | 0.035(0.012-0.076) | 0.007(0.002-0.018) |  |
|  | Global (N= 15) | 0.024(0.017-0.031) | 0.011(0.008-0.015) |  |
| Weir and Cockerham’s *Ɵ_ST_* | Global (N= 15) | 0.007(0.004-0.010) | 0.003(0.001-0.005) |  |
