# Supplementary material for: Molecular pedigree reconstruction and estimation of evolutionary parameters in a wild Atlantic salmon river system with incomplete sampling: a power analysis
Source: BMC Evol Biol. 2014 Mar 31;14:68. doi: 10.1186/1471-2148-14-68 (PMC4021076; doi:10.1186/1471-2148-14-68)
Supplement: Additional file 6 — Assignment success in simulations (medians and standard deviation). [file 1471-2148-14-68-S6.docx]

**Additional file 6. Assignment success in simulations (medians and standard deviation).**

**Additional file 6. Table 1:** Proportion of correctly assigned offspring in simulations with various number of confidence threshold and number of loci used. Results with no error rate and no missing genotyping are also presented. Numbers are median proportions of 100 simulations and standard deviation is given in parentheses.

| Simulations | | Parent offspring cohort pair | | | | | |
| --- | --- | --- | --- | --- | --- | --- | --- |
| Confidence threshold (%) | Number of loci | 77'-81' | 78'-82' | 79'-83' | 80'-84' | 81'-85' | 82'-86' |
| 20 | 5 | 0.00(0.011) | 0.012(0.029) | 0.00(0.013) | 0.00(0.019) | 0.018(0.036) | 0.00(0.041) |
| 20 | 10 | 0.16(0.11) | 0.39(0.15) | 0.19(0.12) | 0.27(0.15) | 0.50(0.15) | 0.41(0.18) |
| 20 | 14 | 0.51(0.17) | 0.74(0.11) | 0.53(0.14) | 0.66(0.15) | 0.81(0.10) | 0.77(0.15) |
| 20 | 20 | 0.82(0.097) | 0.91(0.038) | 0.85(0.075) | 0.89(0.064) | 0.95(0.034) | 0.95(0.061) |
| 20 | 28 | 0.91(0.044) | 0.93(0.028) | 0.94(0.021) | 0.96(0.026) | 0.97(0.012) | 1.00(0.035) |
| 20 | 50 | 0.95(0.04) | 0.94(0.032) | 0.97(0.02) | 0.96(0.019) | 0.98(0.0092) | 1.00(0.026) |
| 40 | 5 | 0.018(0.037) | 0.071(0.084) | 0.017(0.034) | 0.019(0.054) | 0.076(0.085) | 0.045(0.084) |
| 40 | 10 | 0.32(0.16) | 0.61(0.14) | 0.35(0.15) | 0.46(0.17) | 0.67(0.13) | 0.59(0.17) |
| 40 | 14 | 0.66(0.15) | 0.84(0.081) | 0.70(0.12) | 0.79(0.12) | 0.88(0.071) | 0.86(0.11) |
| 40 | 20 | 0.89(0.068) | 0.92(0.03) | 0.90(0.053) | 0.94(0.042) | 0.96(0.022) | 0.95(0.042) |
| 40 | 28 | 0.93(0.041) | 0.93(0.027) | 0.94(0.019) | 0.96(0.024) | 0.98(0.011) | 1.00(0.033) |
| 40 | 50 | 0.95(0.04) | 0.94(0.032) | 0.97(0.02) | 0.96(0.019) | 0.98(0.0092) | 1.00(0.026) |
| 60 | 5 | 0.036(0.071) | 0.15(0.12) | 0.049(0.057) | 0.073(0.092) | 0.14(0.11) | 0.12(0.13) |
| 60 | 10 | 0.43(0.16) | 0.69(0.11) | 0.46(0.15) | 0.58(0.15) | 0.73(0.11) | 0.69(0.15) |
| 60 | 14 | 0.74(0.14) | 0.86(0.061) | 0.75(0.10) | 0.83(0.098) | 0.90(0.058) | 0.88(0.094) |
| 60 | 20 | 0.89(0.058) | 0.93(0.026) | 0.92(0.043) | 0.94(0.035) | 0.96(0.019) | 0.96(0.041) |
| 60 | 28 | 0.93(0.04) | 0.93(0.027) | 0.95(0.018) | 0.96(0.024) | 0.98(0.01) | 1.00(0.032) |
| 60 | 50 | 0.95(0.04) | 0.94(0.032) | 0.97(0.02) | 0.96(0.019) | 0.98(0.0092) | 1.00(0.026) |
| 80 | 5 | 0.078(0.088) | 0.24(0.14) | 0.081(0.081) | 0.12(0.11) | 0.21(0.12) | 0.15(0.15) |
| 80 | 10 | 0.47(0.15) | 0.72(0.096) | 0.51(0.13) | 0.60(0.14) | 0.76(0.094) | 0.71(0.13) |
| 80 | 14 | 0.75(0.12) | 0.87(0.055) | 0.78(0.092) | 0.83(0.093) | 0.90(0.054) | 0.87(0.089) |
| 80 | 20 | 0.89(0.058) | 0.93(0.025) | 0.92(0.04) | 0.94(0.035) | 0.96(0.019) | 0.96(0.04) |
| 80 | 28 | 0.93(0.039) | 0.93(0.027) | 0.95(0.017) | 0.96(0.023) | 0.98(0.01) | 1.00(0.032) |
| 80 | 50 | 0.95(0.04) | 0.94(0.032) | 0.97(0.02) | 0.96(0.019) | 0.98(0.0092) | 1.00(0.026) |
| 95 | 5 | 0.079(0.092) | 0.28(0.14) | 0.099(0.086) | 0.12(0.12) | 0.26(0.12) | 0.17(0.15) |
| 95 | 10 | 0.47(0.15) | 0.72(0.093) | 0.51(0.13) | 0.60(0.14) | 0.76(0.092) | 0.71(0.13) |
| 95 | 14 | 0.75(0.12) | 0.87(0.054) | 0.78(0.092) | 0.83(0.093) | 0.90(0.053) | 0.87(0.09) |
| 95 | 20 | 0.89(0.058) | 0.93(0.025) | 0.92(0.04) | 0.94(0.034) | 0.96(0.019) | 0.96(0.04) |
| 95 | 28 | 0.93(0.039) | 0.93(0.027) | 0.95(0.017) | 0.96(0.023) | 0.98(0.01) | 1.00(0.032) |
| 95 | 50 | 0.95(0.04) | 0.94(0.032) | 0.97(0.02) | 0.96(0.019) | 0.98(0.0092) | 1.00(0.026) |
| Simulations with no missing genotypes | | | |  |  |  |  |
| Confidence threshold | Number of loci | 77'-81' | 78'-82' | 79'-83' | 80'-84' | 81'-85' | 82'-86' |
| 20 | 5 | 0.00(0.022) | 0.024(0.044) | 0.00(0.016) | 0.00(0.021) | 0.03(0.05) | 0.00(0.041) |
| 20 | 10 | 0.28(0.16) | 0.50(0.16) | 0.29(0.15) | 0.33(0.15) | 0.62(0.16) | 0.50(0.18) |
| 20 | 14 | 0.67(0.17) | 0.81(0.095) | 0.67(0.14) | 0.70(0.14) | 0.88(0.092) | 0.82(0.13) |
| 20 | 20 | 0.89(0.056) | 0.93(0.034) | 0.92(0.053) | 0.92(0.063) | 0.96(0.027) | 0.95(0.05) |
| 20 | 28 | 0.93(0.047) | 0.93(0.03) | 0.95(0.021) | 0.96(0.025) | 0.97(0.012) | 1.00(0.031) |
| 20 | 50 | 0.91(0.058) | 0.92(0.035) | 0.95(0.022) | 0.96(0.022) | 0.98(0.012) | 1.00(0.031) |
| 40 | 5 | 0.018(0.059) | 0.11(0.10) | 0.025(0.046) | 0.038(0.059) | 0.093(0.11) | 0.045(0.099) |
| 40 | 10 | 0.48(0.19) | 0.69(0.13) | 0.47(0.17) | 0.55(0.17) | 0.76(0.14) | 0.67(0.15) |
| 40 | 14 | 0.79(0.12) | 0.88(0.061) | 0.80(0.11) | 0.81(0.11) | 0.92(0.06) | 0.91(0.095) |
| 40 | 20 | 0.93(0.04) | 0.94(0.024) | 0.94(0.035) | 0.94(0.042) | 0.97(0.016) | 1.00(0.043) |
| 40 | 28 | 0.93(0.047) | 0.93(0.03) | 0.95(0.019) | 0.96(0.023) | 0.97(0.012) | 1.00(0.03) |
| 40 | 50 | 0.91(0.058) | 0.92(0.035) | 0.95(0.022) | 0.96(0.022) | 0.98(0.012) | 1.00(0.031) |
| 60 | 5 | 0.055(0.096) | 0.21(0.15) | 0.061(0.077) | 0.093(0.094) | 0.19(0.14) | 0.13(0.13) |
| 60 | 10 | 0.59(0.17) | 0.77(0.099) | 0.57(0.16) | 0.64(0.14) | 0.81(0.11) | 0.74(0.12) |
| 60 | 14 | 0.83(0.10) | 0.90(0.051) | 0.85(0.084) | 0.84(0.085) | 0.94(0.051) | 0.91(0.075) |
| 60 | 20 | 0.95(0.035) | 0.94(0.023) | 0.95(0.029) | 0.94(0.034) | 0.97(0.014) | 1.00(0.039) |
| 60 | 28 | 0.93(0.047) | 0.93(0.03) | 0.95(0.019) | 0.96(0.021) | 0.97(0.012) | 1.00(0.03) |
| 60 | 50 | 0.91(0.058) | 0.92(0.035) | 0.95(0.022) | 0.96(0.022) | 0.98(0.012) | 1.00(0.031) |
| 80 | 5 | 0.12(0.11) | 0.31(0.15) | 0.11(0.096) | 0.16(0.12) | 0.28(0.15) | 0.21(0.16) |
| 80 | 10 | 0.62(0.16) | 0.79(0.086) | 0.61(0.14) | 0.64(0.13) | 0.83(0.098) | 0.76(0.11) |
| 80 | 14 | 0.83(0.096) | 0.90(0.048) | 0.85(0.076) | 0.84(0.072) | 0.94(0.047) | 0.91(0.072) |
| 80 | 20 | 0.95(0.034) | 0.95(0.021) | 0.95(0.028) | 0.94(0.031) | 0.97(0.014) | 1.00(0.039) |
| 80 | 28 | 0.93(0.047) | 0.93(0.03) | 0.95(0.019) | 0.96(0.021) | 0.97(0.012) | 1.00(0.03) |
| 80 | 50 | 0.91(0.058) | 0.92(0.035) | 0.95(0.022) | 0.96(0.022) | 0.98(0.012) | 1.00(0.031) |
| 95 | 5 | 0.13(0.11) | 0.33(0.15) | 0.12(0.10) | 0.17(0.12) | 0.32(0.14) | 0.20(0.16) |
| 95 | 10 | 0.62(0.16) | 0.79(0.084) | 0.61(0.14) | 0.64(0.13) | 0.83(0.095) | 0.76(0.12) |
| 95 | 14 | 0.83(0.096) | 0.90(0.048) | 0.85(0.076) | 0.84(0.072) | 0.94(0.047) | 0.91(0.072) |
| 95 | 20 | 0.95(0.034) | 0.95(0.021) | 0.95(0.028) | 0.94(0.031) | 0.97(0.014) | 1.00(0.039) |
| 95 | 28 | 0.93(0.047) | 0.93(0.03) | 0.95(0.019) | 0.96(0.021) | 0.97(0.012) | 1.00(0.03) |
| 95 | 50 | 0.91(0.058) | 0.92(0.035) | 0.95(0.022) | 0.96(0.022) | 0.98(0.012) | 1.00(0.031) |
| Simulations with no genotyping error rate | | | | |  |  |  |
| Confidence threshold | Number of loci | 77'-81' | 78'-82' | 79'-83' | 80'-84' | 81'-85' | 82'-86' |
| 20 | 5 | 0.00(0.023) | 0.018(0.037) | 0.00(0.012) | 0.00(0.03) | 0.033(0.049) | 0.00(0.039) |
| 20 | 10 | 0.24(0.15) | 0.52(0.16) | 0.25(0.14) | 0.33(0.18) | 0.58(0.16) | 0.50(0.19) |
| 20 | 14 | 0.65(0.18) | 0.84(0.11) | 0.65(0.15) | 0.77(0.14) | 0.89(0.10) | 0.89(0.12) |
| 20 | 20 | 0.95(0.072) | 0.97(0.021) | 0.93(0.055) | 0.96(0.036) | 0.98(0.022) | 1.00(0.019) |
| 20 | 28 | 1.00(0.0092) | 0.98(0.0035) | 0.99(0.0089) | 0.98(0.0045) | 0.99(0.002) | 1.00(0.0043) |
| 20 | 50 | 1.00(0.00) | 0.98(0.00) | 0.99(0.00) | 0.98(0.00) | 0.99(0.00) | 1.00(0.00) |
| 40 | 5 | 0.027(0.051) | 0.095(0.093) | 0.025(0.032) | 0.038(0.067) | 0.10(0.097) | 0.045(0.10) |
| 40 | 10 | 0.44(0.19) | 0.73(0.14) | 0.43(0.17) | 0.57(0.18) | 0.74(0.14) | 0.74(0.17) |
| 40 | 14 | 0.81(0.15) | 0.92(0.066) | 0.78(0.12) | 0.87(0.092) | 0.94(0.065) | 0.95(0.073) |
| 40 | 20 | 0.96(0.041) | 0.98(0.013) | 0.97(0.032) | 0.98(0.024) | 0.98(0.014) | 1.00(0.017) |
| 40 | 28 | 1.00(0.009) | 0.98(0.0039) | 0.99(0.0064) | 0.98(0.0047) | 0.99(0.0023) | 1.00(0.0043) |
| 40 | 50 | 1.00(0.00) | 0.98(0.00) | 0.99(0.00) | 0.98(0.00) | 0.99(0.00) | 1.00(0.00) |
| 60 | 5 | 0.068(0.078) | 0.20(0.13) | 0.057(0.057) | 0.077(0.10) | 0.19(0.12) | 0.091(0.14) |
| 60 | 10 | 0.58(0.18) | 0.78(0.11) | 0.55(0.16) | 0.66(0.15) | 0.81(0.12) | 0.80(0.14) |
| 60 | 14 | 0.84(0.12) | 0.92(0.05) | 0.82(0.09) | 0.88(0.068) | 0.94(0.05) | 0.95(0.061) |
| 60 | 20 | 0.96(0.039) | 0.97(0.015) | 0.96(0.029) | 0.96(0.022) | 0.98(0.015) | 1.00(0.025) |
| 60 | 28 | 1.00(0.01) | 0.98(0.0041) | 0.99(0.0091) | 0.98(0.005) | 0.99(0.0027) | 1.00(0.0075) |
| 60 | 50 | 1.00(0.00) | 0.98(0.00) | 0.99(0.00) | 0.98(0.00) | 0.99(0.00) | 1.00(0.00) |
| 80 | 5 | 0.12(0.095) | 0.31(0.14) | 0.11(0.078) | 0.15(0.12) | 0.28(0.13) | 0.17(0.17) |
| 80 | 10 | 0.62(0.15) | 0.78(0.089) | 0.60(0.13) | 0.70(0.13) | 0.81(0.098) | 0.82(0.13) |
| 80 | 14 | 0.85(0.10) | 0.91(0.049) | 0.84(0.082) | 0.88(0.065) | 0.94(0.047) | 0.95(0.063) |
| 80 | 20 | 0.96(0.04) | 0.97(0.016) | 0.96(0.03) | 0.96(0.025) | 0.98(0.016) | 1.00(0.027) |
| 80 | 28 | 1.00(0.01) | 0.98(0.0047) | 0.99(0.0098) | 0.98(0.005) | 0.99(0.0033) | 1.00(0.0075) |
| 80 | 50 | 1.00(0.00) | 0.98(0.00) | 0.99(0.00) | 0.98(0.00) | 0.99(0.00) | 1.00(0.00) |
| 95 | 5 | 0.13(0.10) | 0.34(0.14) | 0.13(0.084) | 0.17(0.12) | 0.32(0.12) | 0.17(0.17) |
| 95 | 10 | 0.63(0.15) | 0.79(0.086) | 0.60(0.13) | 0.70(0.13) | 0.82(0.095) | 0.82(0.12) |
| 95 | 14 | 0.85(0.10) | 0.91(0.05) | 0.83(0.082) | 0.88(0.065) | 0.94(0.047) | 0.95(0.063) |
| 95 | 20 | 0.96(0.041) | 0.97(0.016) | 0.96(0.029) | 0.96(0.025) | 0.98(0.016) | 1.00(0.027) |
| 95 | 28 | 1.00(0.01) | 0.98(0.0047) | 0.99(0.0098) | 0.98(0.005) | 0.99(0.0033) | 1.00(0.0075) |
| 95 | 50 | 1.00(0.00) | 0.98(0.00) | 0.99(0.00) | 0.98(0.00) | 0.99(0.00) | 1.00(0.00) |
