# Supplementary material for: Molecular pedigree reconstruction and estimation of evolutionary parameters in a wild Atlantic salmon river system with incomplete sampling: a power analysis
Source: BMC Evol Biol. 2014 Mar 31;14:68. doi: 10.1186/1471-2148-14-68 (PMC4021076; doi:10.1186/1471-2148-14-68)
Supplement: Additional file 7 — Additional pedigree statistics and figures. [file 1471-2148-14-68-S7.pdf]

Additional file 7. Additional pedigree statistics and figures.

Additional file 7. Table: Empirical pedigree statistics with 80% confidence threshold. Statistics are given for six parent – offspring cohorts and the combined pedigree using 14 microsatellite loci and 80% confidence threshold for all cohorts and combined pedigree, and also with 28 microsatellite markers for the two parent – offspring cohorts (i.e. ‘77 – ‘81 and ‘79 – ‘83, listed in parentheses).

	77'-81' (28 loci)[1]	78'-82'	79'-83' (28 loci)[1]	80'-84'	81'-85'	82'-86'	Cohorts combined[2]
Samples available[1]							
Candidate mothers	120 (120)	61	193 (193)	111	135	129	749
Candidate fathers	22(22)	6	74(74)	13	17	20	152
Offspring	145(151)	150	201(105)	118	213	49	876
Total	287(293)	217	468 (472)	242	365	198	1482
Offspring with parent(s) identified							
Maternal links identified	48(52)	74	80(95)	42	108	18	370
Paternal links identified	3(3)	0	15 (21)	4	26	2	50
Offspring with at least one parent identified	51(55)	74	90(111)	44	114	18	391
Offspring with both parents identified	0 (0)	0	5(5)	2	20	2	29
Maternal half sib families[4]							
Total family number	11 (11)	17	16 (18)	8	29	1	82
Mean family size	2.6 (2.8)	3.4	2.9(3.0)	2.6	2.6	2.0	2.8
Maximum family size	5 (7)	7	8(8)	4	5	2	8
Paternal half sib families[4]							
Total family number	1 (1)	0	2 (4)	1	6	0	10
Mean family size	3 (3)	0	2 (2.5)	2	3.7	0	3.1
Maximum family size	3 (3)	0	2 (3)	2	7	0	7
Other							
Full-sib families[4]	0 (0)	0	0	0	4	0	4
Two generational links							30

1- Successfully genotyped individuals that were included in the parentage analysis (see also: Table 1)
2- The total number of individuals with 28 loci is slightly more than that of with 14 loci, as fewer individuals were filtered out due to the minimum number of loci genotyped (N <7) threshold.
3- Based on 14 loci.
4- Includes only families with more than 1 offspring identified

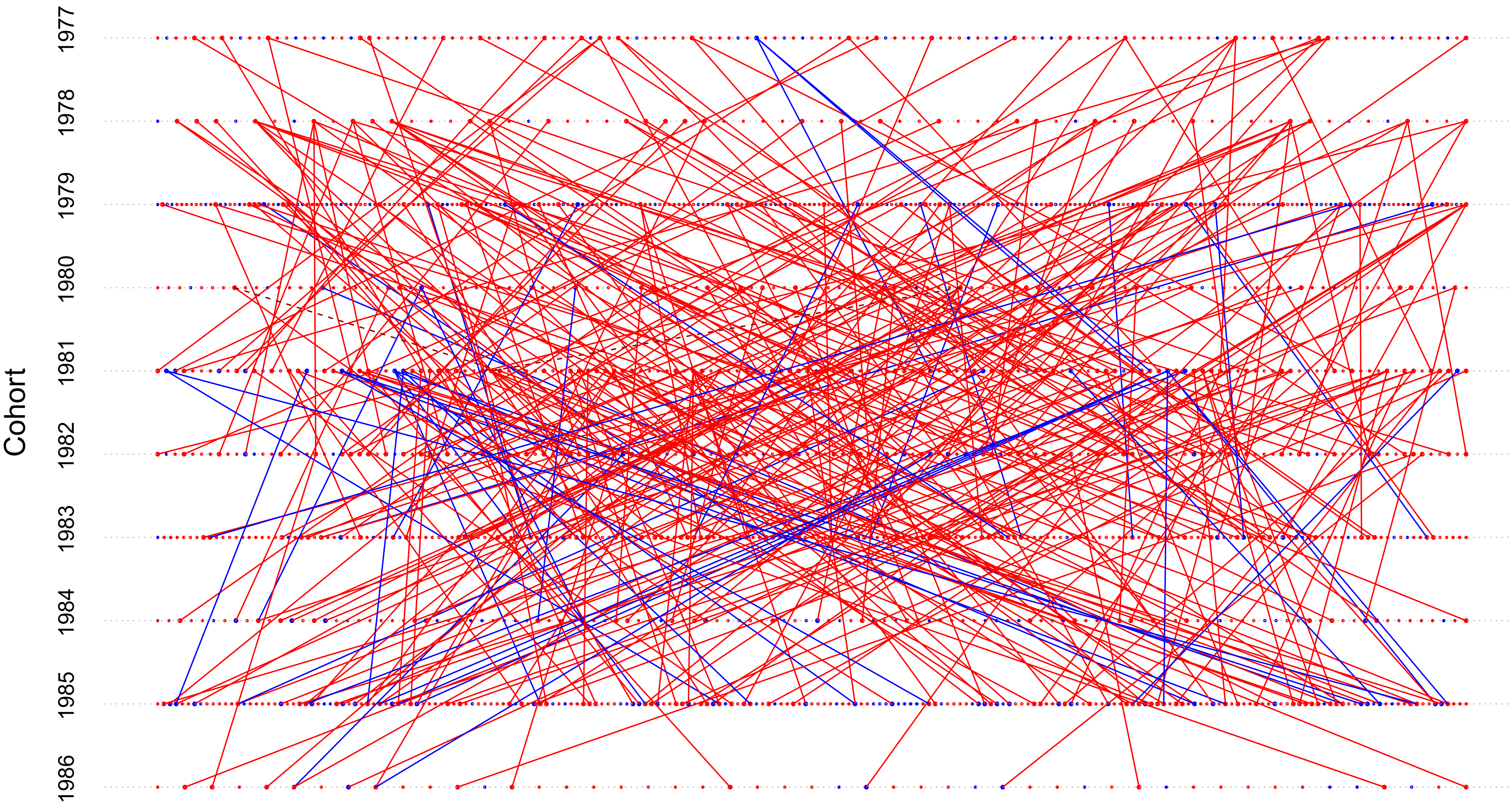

Additional file 7. Figure 1: Illustration of links between parents and progeny across all cohorts. The parentage links are based on the 0.95 probability threshold. Red and blue dots are female and male individuals, and red and blue lines are maternal and paternal parentage links, respectively. Individuals with a parent-offspring link are shown in larger size dots, compared to individuals in the dataset with no links identified. Two multi-spawner females that had offspring in two consecutive years (1980 and 1981) are connected with a black dashed line.

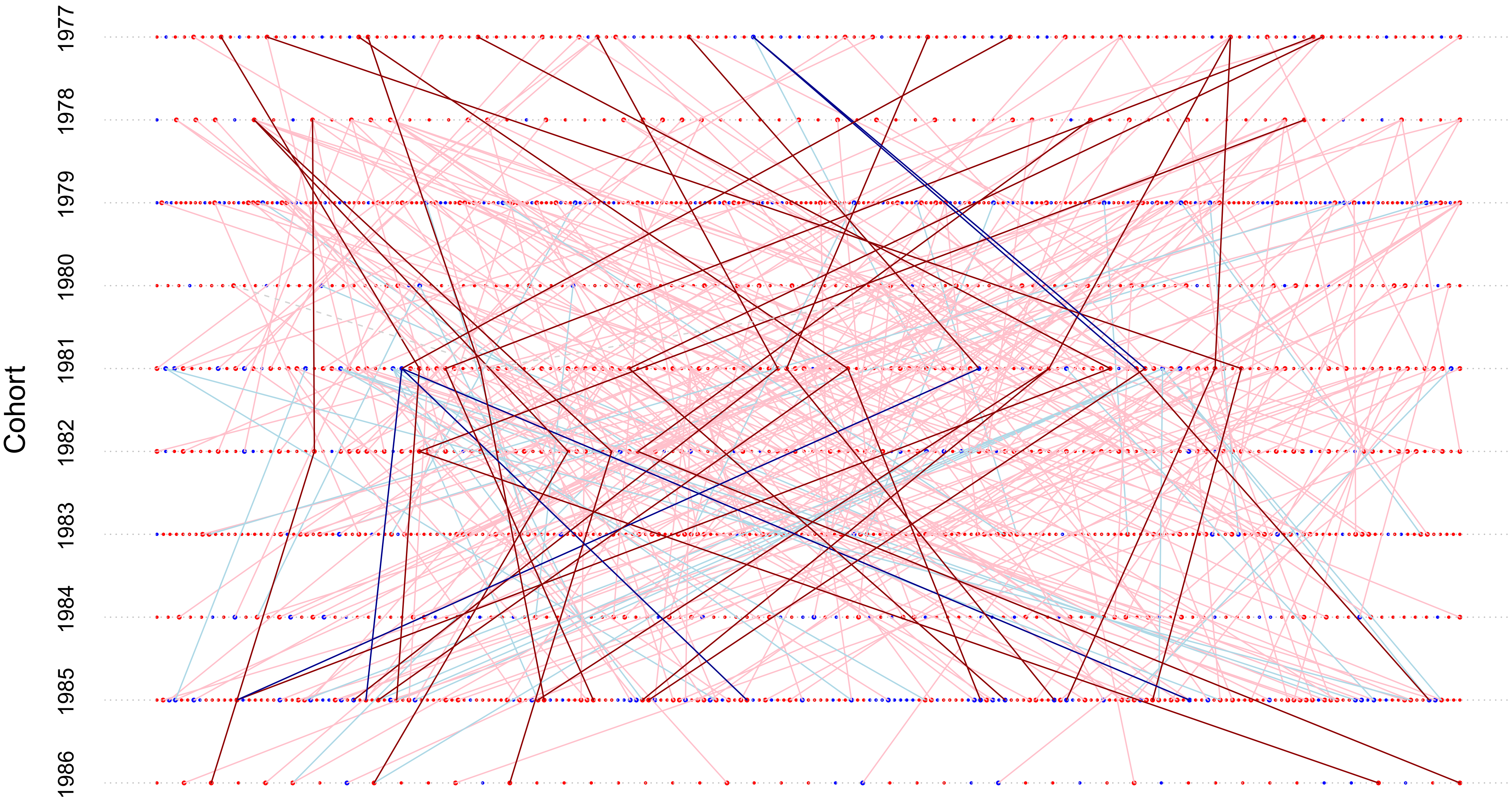

Additional file 7. Figure 2. Illustration of the links between parents and progeny across all cohorts with two generational links emphasized. The figure is same as above except individuals with two generational links are highlighted with darker colours (i.e. dark red and dark blue for female and male individuals respectively).

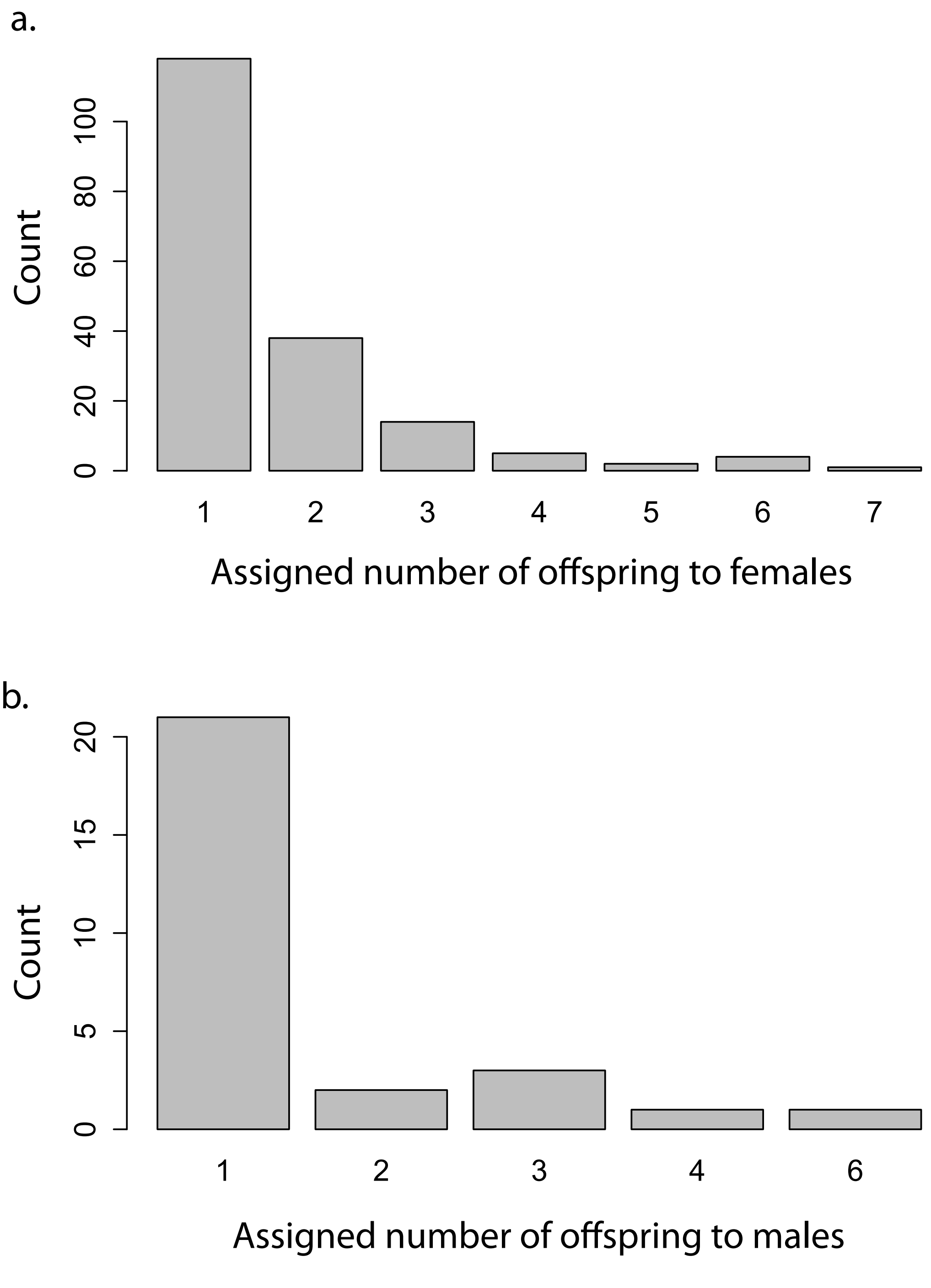

Additional file. Figure 3: Distribution of assigned offspring number among (a) female and, (b) male parents.
