# Supplementary material for: Molecular pedigree reconstruction and estimation of evolutionary parameters in a wild Atlantic salmon river system with incomplete sampling: a power analysis
Source: BMC Evol Biol. 2014 Mar 31;14:68. doi: 10.1186/1471-2148-14-68 (PMC4021076; doi:10.1186/1471-2148-14-68)
Supplement: Additional file 8 — Power to detect relative reproductive success among groups of fish based on Burrishoole Atlantic salmon demography and sampling information, additional figures. [file 1471-2148-14-68-S8.pdf]

Additional file 8. Power to detect relative reproductive success among groups of fish based on Burrishoole Atlantic salmon demography and sampling information, additional figures.

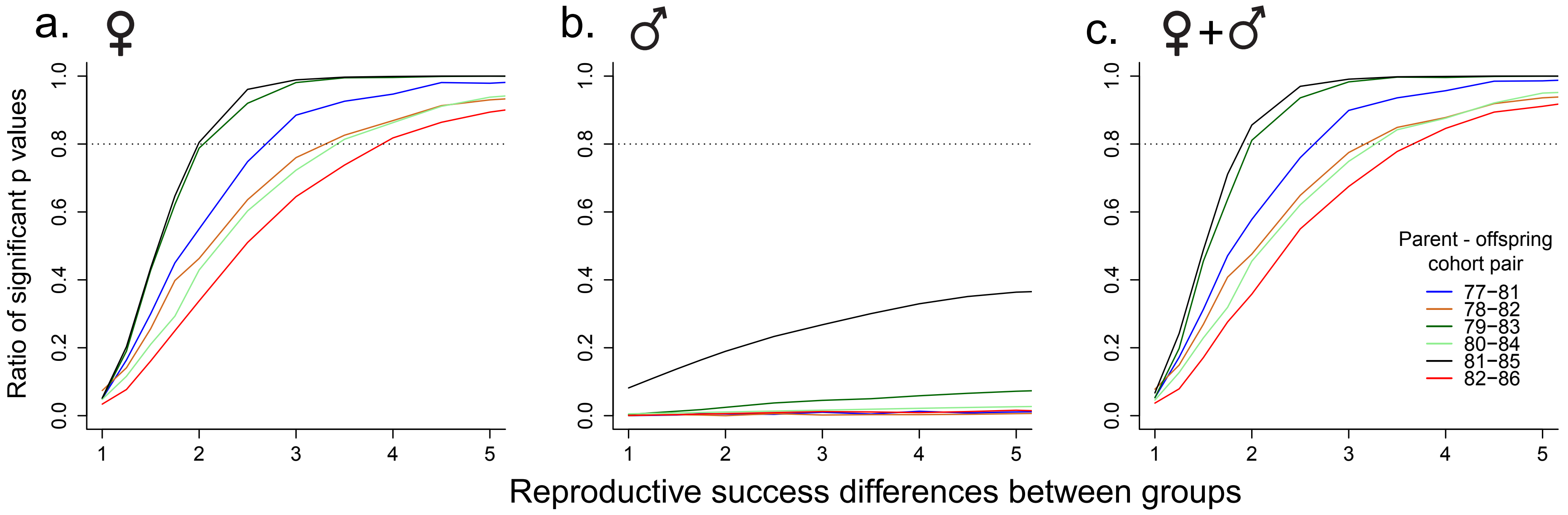

Additional file 8. Figure 1. Power to detect relative reproductive success among groups of fish based on Burrishoole Atlantic salmon demography and sampling information when the groups are equal (0.5). Each line is the power curve of a particular parent-offspring cohort pair for females (a), males (b) and both sexes together (c), respectively.

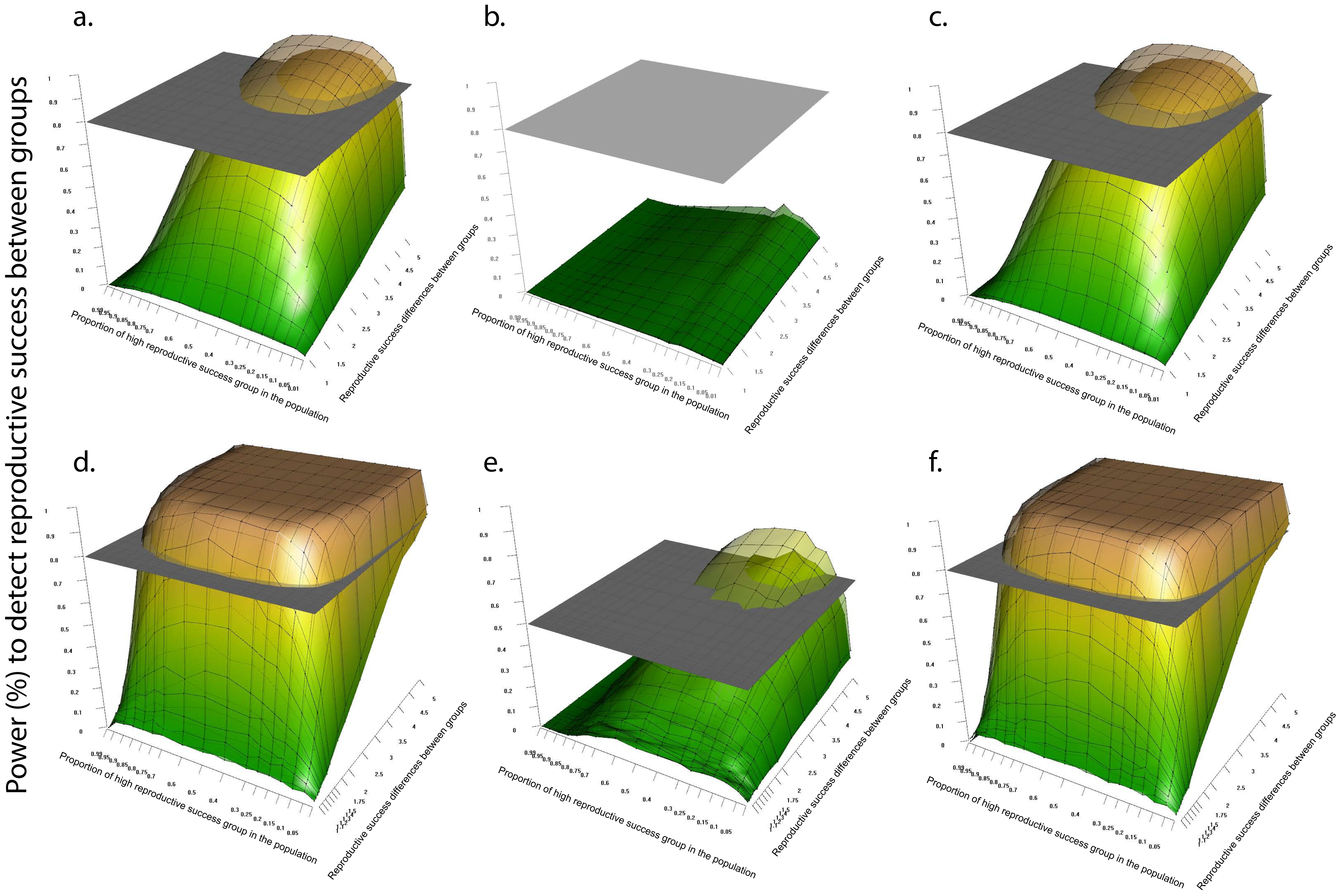

Additional file 8. Figure 2. Power to detect relative reproductive success among groups of fish based on Burrishoole Atlantic salmon demography and sampling information, along different group size proportion between high rand low reproductive success groups. Panel a-c are the average (mean) power graphs when cohorts' information are calculated separately, for female (a), male (b) and when sex information is combined (c). Panels d-e are the power graphs when all cohorts' information are combined, for female (d), male (a) and when sex information is combined (e). The gray plane parallel to xy plane is the 80% detection threshold, except in panel e, which shows the 50% power threshold. The tick marks on the xy plane and the mesh lines on power surface are identical, which are also equal to values used in the simulations. Note that the tick marks are denser at lower values of reproductive success differences at panels c and d (i.e. cohorts combined), which required finer resolution at low difference values. The outer shell of the surface graph, which is transparent, presents results when parentage is resolved with 28 loci and the inner shell is the results when parentage is resolved with 14 loci.
