# Supplementary material for: Molecular pedigree reconstruction and estimation of evolutionary parameters in a wild Atlantic salmon river system with incomplete sampling: a power analysis
Source: BMC Evol Biol. 2014 Mar 31;14:68. doi: 10.1186/1471-2148-14-68 (PMC4021076; doi:10.1186/1471-2148-14-68)

Additional file 9. Ranges of heritability estimates for a given heritability value across cohorts.

Additional file 9. Figure : Ranges of heritability estimates for a given heritability value in (a) 1977-1981, (b) 1979-1983 parent-offspring cohort pairs and (c) the combined cohort estimate using pedigrees that were resolved at 28 loci (gray contours) and 14 loci (pink contours). Boxes and lines show the 25th - 75th and 5th - 95th percentiles of the estimated range..

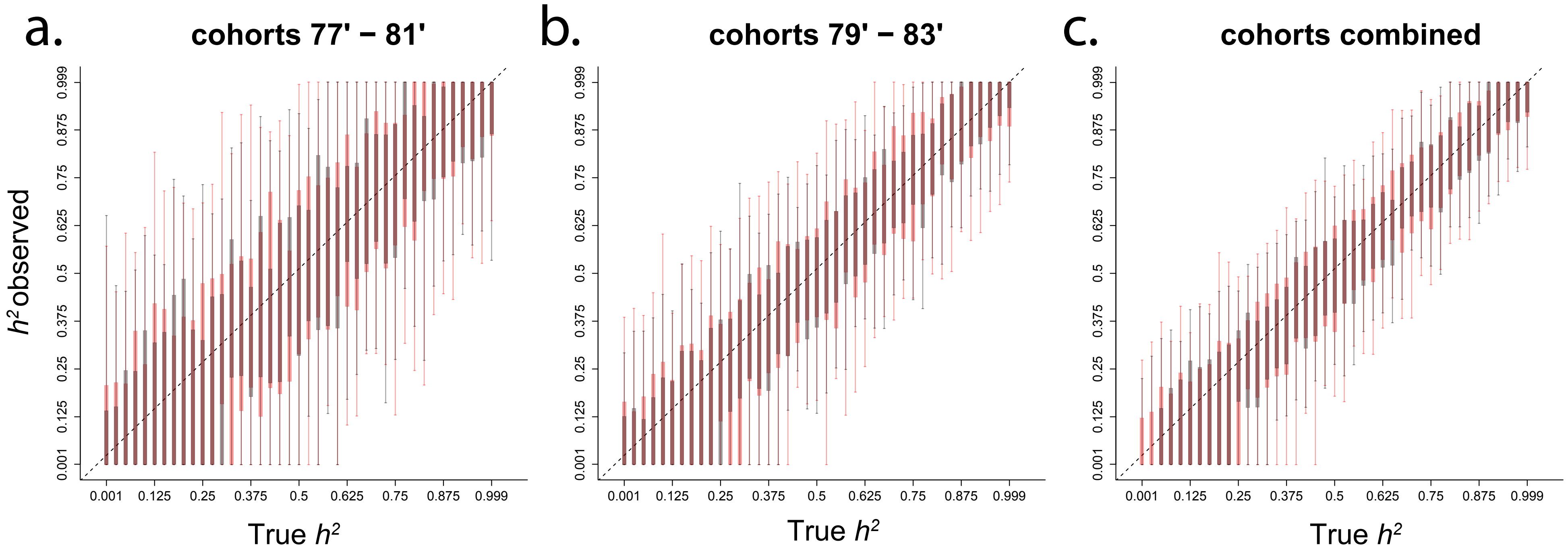

Supplement: Additional file 9 — Ranges of heritability estimates for a given heritability value. [file 1471-2148-14-68-S9.pdf]
